# Induction of mitochondria-mediated apoptosis and suppression of tumor growth in zebrafish xenograft model by cyclic dipeptides identified from *Exiguobacterium acetylicum*

**DOI:** 10.1038/s41598-020-70516-x

**Published:** 2020-08-13

**Authors:** Sekar Jinendiran, Weilin Teng, Hans-Uwe Dahms, Wangta Liu, Vinoth Kumar Ponnusamy, Charles Chien-Chih Chiu, B. S. Dileep Kumar, Natesan Sivakumar

**Affiliations:** 1 Department of Molecular Microbiology, School of Biotechnology, Madurai Kamaraj University, Madurai, 625021 India; 2 Department of Biotechnology, Kaohsiung Medical University, Kaohsiung, 80708 Taiwan; 3 Department of Biomedical Science and Environmental Biology, Kaohsiung Medical University, Kaohsiung, 80708 Taiwan; 4 Department of Marine Biotechnology and Bioresources, National Sun Yat-Sen University, Kaohsiung, 80424 Taiwan; 5 Department of Medicinal Applied Chemistry, Kaohsiung Medical University, Kaohsiung, 80708 Taiwan; 6 Agro-Processing and Technology Division, CSIR-National Institute for Interdisciplinary Science and Technology, Thiruvananthapuram, 695019 India

**Keywords:** Cancer, Microbiology, Molecular biology, Biomarkers, Molecular medicine

## Abstract

Colorectal cancer is the most common type of gastrointestinal cancers with poor survival and limited therapeutic options. In this study, four structurally different cyclic dipeptides (or diketopiperazine) were isolated and identified as cyclo (l-Pro-l-Leu), cyclo (l-Pro-l-Val), cyclo (l-Pro-l-Phe) and cyclo (l-Pro-l-Tyr) from the ethyl acetate extract in the cell-free filtrate of *Exiguobacterium acetylicum* S01. The anticancer potential of identified DKPs on colorectal cancer HT-29 cells in vitro and in vivo zebrafish xenograft model was evaluated. The MTT (3-(4, 5-dimethylthiazol-2yl)-2, 5-diphenyltetrazolium bromide)) assay showed that four DKPs exhibited significant inhibition of HT-29 cells viability in a dose-dependent manner whereas there were no cytotoxic effects on normal mouse fibroblast 3T3 cells. Also, we observed that all DKPs induce early and late apoptotic cell death in HT-29 cells. Moreover, the expression levels of apoptotic (cytochrome-c, caspase-3 and Bid) and anti-apoptotic (Bcl-2) markers were up- and down-regulated in HT-29 cells in response to DKPs treatments. Furthermore, these four DKPs remarkably inhibited the tumor progression in a zebrafish xenograft model within a nonlethal dose range. Overall, our findings suggest that cyclic dipeptides derived from *E. acetylicum* S01 could be promising chemopreventive/ therapeutic candidates against cancer.

## Introduction

Colorectal cancer (CRC) is the third most commonly diagnosed malignancy and the second leading cause of cancer-related deaths in the world. According to the World Health Organization reports, the global burden of CRC is expected to increase by 60% i.e., 2.2 million new cases and 1.1 million deaths by the year 2030^1^. The risk factor for developing CRC is connected with age, genetic predisposition, disease history, and lifestyle including food habits. Also, the development of tumor proceeds through a series of genetic and epigenetic alterations involving the activation of oncogenes and inactivation of tumor suppressor genes^1,2^. The progressive inhibition of apoptosis in CRC has been found during the cellular transformation of the normal intestinal epithelium to adenocarcinomas^3^, which contribute to promoting neoplastic progression and resistance to anticancer drugs^4^. However, 5-fluorouracil (5-FU)-based chemotherapy is the most common treatments for the early stage of CRC^4^. At the metastatic stage, the cancer is generally unresponsive to available therapies and thus is commonly fatal^2^. Therefore, there is a need to develop a novel therapeutic approach that is more effective than currently used treatment regimens. In this perspective, the use of cyclic peptides is receiving considerable attention as an alternative therapeutic agent against CRC^5,6^.

Cyclic dipeptides (or diketopiperazine—DKP) are organic substances formed by two amino acids joined by covalent bonds known as amide or peptide bonds. These dipeptides are an important class of biologically active molecules. They are produced by a wide range of organism encompassing both Gram-positive and Gram-negative bacteria, fungi, sponges, and animals^7^. Several cyclic dipeptides have diverse biological functions, such as antimicrobial, antifungal, antiviral, anti-inflammatory, anticancer, and immunosuppressive activities^8,9^. Nishanth Kumar and co-workers reported that cyclo (d-Tyr-d-Phe) isolated from *Bacillus* sp, which induces apoptosis by the activation of caspase-3 in a pulmonary adenocarcinoma cell line^10^. Another report established that cyclo (l-Phe-l-His) obtained from *Aspergillus ustus* and cyclo (l-Phe-l-Pro) isolated from the probiotic strain *Lactobacillus plantarum* inhibits the growth of various cancer cells^11,12^. Moreover, earlier studies demonstrated that a crude mixture of DKPs obtained from the *Pseudomonas aeruginosa* PAO1 strain, which promotes apoptotic cell death in HeLa and Caco-2 cells^13^.

In recent years, several studies showed that deregulated proliferation and inhibition of the apoptotic process is a critical factor for all tumor development. Therefore, the regulation of cell growth and apoptosis affords an evident target for therapeutic intervention in all cancers^14^. In general, apoptosis can be induced either by an extrinsic pathway mediated through the activation of death receptors or by an intrinsic signalling pathway, which involves in the alteration of mitochondrial membrane permeability (MMP), thereby inducing the release of cytochrome-c from mitochondria^15,16^. This intrinsic pathway is regulated by the Bcl-2 family of proteins, which controls MMP^17^. Recently, zebrafish is an emerging animal model for examining tumors^18^ and tumor–organ interaction due to many advantages. Thus, zebrafish xenograft model is receiving considerable attention as a reliable whole-animal model system to rapidly screen for small-molecule as drug candidates^19,20^. However, the purification and characterization of DKPs from the potentially probiotic strain *Exiguobacterium acetylicum* S01 and their therapeutic efficacy on CRC have not yet been studied extensively. Hence, the objective of the present investigation was aimed to isolate and identify DKPs from the cell-free extract of *E. acetylicum* S01 and to evaluate their therapeutic potential in colorectal cancer HT-29 cells in vitro and in vivo zebrafish xenograft model.

## Results

### Purification of bioactive metabolites

The oily reddish-yellow extract was obtained from the cell-free culture filtrate of *E. acetylicum* S01. The crude extract was taken for purification of bioactive metabolites using silica gel column chromatography (SGCC) following the method described by Nishanth Kumar et al.^21^ with slight modification. Column chromatography of this extract yielded four crystalline compounds eluted at 45% ethyl acetate in hexane (DKP-1), 55% ethyl acetate in hexane (DKP-2), 70% ethyl acetate in hexane (DKP-3), and 5% methanol in chloroform (DKP-4) respectively. Thin-layer chromatography of the eluted all DKPs in silica gel TLC sheets demonstrated single spots.

### Structural elucidation of bioactive compounds

**Figure 1:**
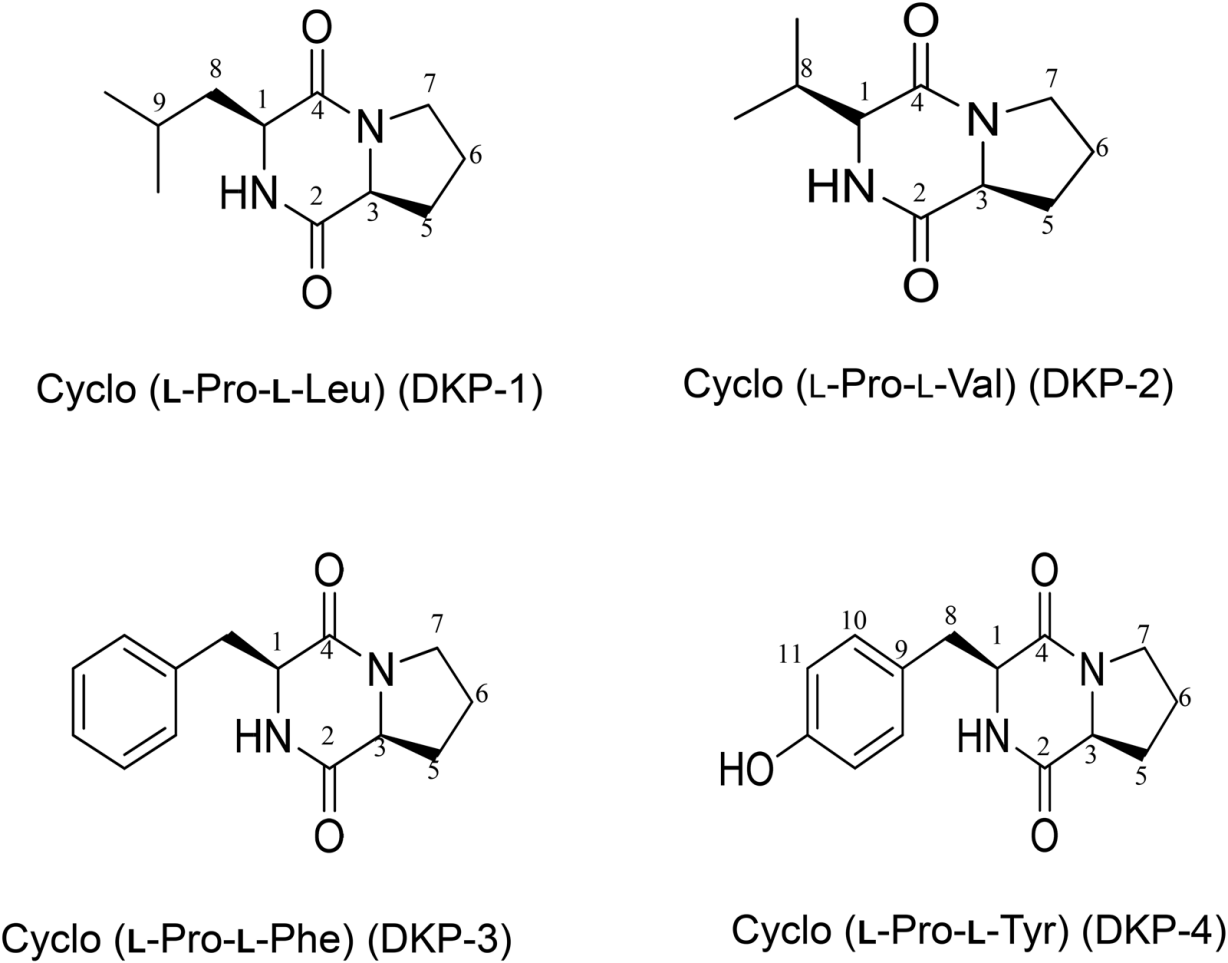
Structures of isolated cyclic dipeptides from the ethyl acetate extract in the cell-free filtrate of *E. acetylicum* S01. Chemical structures of the four cyclic dipeptides identified from *E. acetylicum* S01.

Structures of purified compounds were elucidated by ^1^H and ^13^C NMR and mass spectrometric analysis (HR-ESI–MS). The compounds isolated from *E. acetylicum* S01 were identified as cyclo (l-Pro-l-Leu) (DKP-1), cyclo (l-Pro- l-Val) (DKP-2), cyclo (l-Pro-l-Phe) (DKP-3), and cyclo (l-Pro-l-Tyr) (DKP-4) (Fig. 1). Both ^1^H and ^13^C NMR spectra of all DKPs exhibited a signal at δH 4.13 (t) with *J* value around 8 Hz with a corresponding δC of 59.0, together with multiplets around δH 2.3, 2.0, and 3.5 ppm, which shows the presence of a proline residue. The amide hydrogen signal in ^1^H NMR spectra of all DKPs, and comparison with standard amino acids, and all DKPs presently identified were found to have proline. The absolute configuration was determined by digital polarimeter and by comparison against reported specific rotation values of the respective compound^22–24^.

#### DKP 1: Cyclo (l-Pro-l-Leu)

Yellow crystalline powder 55 mg; TLC (hexane: ethyl acetate, 80:20 v/v): Rf = 0.50; HPLC (methanol (MeOH): acetonitrile (CH_3_CN), 70:30 v/v): RT = 0.96 min; Specific Optical Rotation: [α]^24^_D_ ­124° (*c* = 0.3, ethanol), for R,R [α]^24^_D_ = − 124° (*c* = 0.4, ethanol)^22^; ^1^H and ^13^C NMR data, see Table 1 (Fig. S1A, B); HR-ESI–MS (m/z): [M + H]^+^ calcd for C_11_H_19_N_2_O_2_, 211.1450; found, 211.1446 (Fig. S2A).

**Table 1:** NMR data for DKPs 1 – 4 in CDCl_3_ (multi, *J,* Hz).

Carbon No	DKP-1		DKP-2		DKP-3		DKP-4	
	δH	δC	δH	δC	δH	δC	δ**H**	δ**C**
1	4.02, dd (7)	53.4	3.87 (s)	60.4	4.20, dd (10.5, 3)	56.1	4.20, dd (3.5, 11.5)	56.2
OH							4.15, dd (9.5, 2.5)	
NH	6.21 (s)		6.19 (s)		5.57 (s)		5.93 (s)	
2		166.2		164.9		165.0		165.2
3	4.13, t (8.0)	58.9	4.01, app t, (8)	58.8	4.01, t (7.5)	59.1	4.0, td (5.5, 7.5)	59.1
4		170.2		170.1		169.3		169.7
5	2.33, 2.37, m	28.1	2.28, 2.33, m	28.4	1.93, 1.96, 2.24, 2.29, m	28.3	1.96, 2.27, m	28.3
6	2.02, 2.18, m	24.6	1.93, 2.02, m	22.3	1.18, 1.85, m	22.5	1.89, m	22.4
7	3.52, 3.64, m	45.5	3.49, 3.60, m	45.1	3.59, m	45.4	3.50, 3.59, m	45.4
8	1.17, 1.82, 1.88, 1.94, m	38.6	1.17, 1.88, 1.93, 2.02, m	28.5	2.71, dd (14.5, 10.5)	36.8	2.71, dd (14.5, 9.5), 3.37, dd (14.5, 4)	35.9
9	1.54, ddd (14.5, 9.5, 5)	23.2	2.54, ddd (2.5)	19.1				126.8
10	1.01, d (6.5)	22.7	1.01, d (7)	19.0			6.97, d (8.5 H10,10′)	130.3
11	0.96, d (6.5)	21.2	0.84, d *(*7)				6.70, d (8.5 H11,11′)	116.1
12								155.7
Ph					7.15–7.30	127.5, 129.1, 129.2, 135.9		

*s* singlet; *d* doublet; *dd* doublet of doublets; *m* multiplet; *t* triplet.

#### DKP 2: Cyclo (l-Pro-l-Val)

Pale yellow crystalline powder 41 mg; TLC (hexane: ethyl acetate, 20:80 v/v): Rf = 0.6; HPLC (MeOH:CH_3_CN, 70:30 v/v): RT = 0.97 min; Specific Optical Rotation: [α]^24^_D_ = − 144° (*c* = 0.3, ethanol), for R,R, [α]^20^_D_ − 139.4° (*c* = 0.16, EtOH)^23^; ^1^H and ^13^C NMR data, see Table 1 (Fig*.* S1C,D); HR-ESI–MS (m/z): [M + H]^+^ calcd for C_10_H_17_N_2_O_2,_ 197.1292; found, 197.1290 (Fig. S2B).

#### DKP 3: Cyclo (l-Pro-l-Phe)

Pale yellow powder (9.5 mg); TLC (hexane: ethyl acetate, 10:90 v/v): Rf = 0.3; HPLC (MeOH:CH_3_CN, 60:40 v/v): RT value 0.86 min; Specific Optical Rotation: [α]^24^_D_ = − 82° (*c* = 0.4, MeoH), for R,R, [α]^25^_D_ = − 92 (*c* = 1.1, MeoH)^24^; ^1^H and ^13^C NMR data, see Table 1 (Fig. S1E,F); HR-ESI–MS (m/z): [M + H]^+^ calcd for C_14_H_17_N_2_O_3,_ 245.1292; found, 245.1290 (Fig. S2C).

#### DKP 4: Cyclo (l-Pro-l-Tyr)

Pale yellow powder (25.5 mg); TLC (methanol: chloroform, 1:99 v/v): Rf = 0.80; HPLC (MeOH:CH_3_CN, 50:50 v/v): RT = 0.99 min; Specific Optical Rotation: α]^24^_D_ = − 106° (*c* = 0.5, MeOH), for R,R,[α]^25^_D_ = − 100°(c = 0.5, MeOH)^24^; ^1^H and ^13^C NMR data, see Table 1 (Fig. S1G,H); HR-ESI–MS (m/z): [M + H]^+^ calcd for C_14_H_17_N_2_O_3_, 261.1241; found, 261.1239 (Fig. S2D).

### Effects of DKPs on cell viability of cancer and normal cells

**Figure 2:**
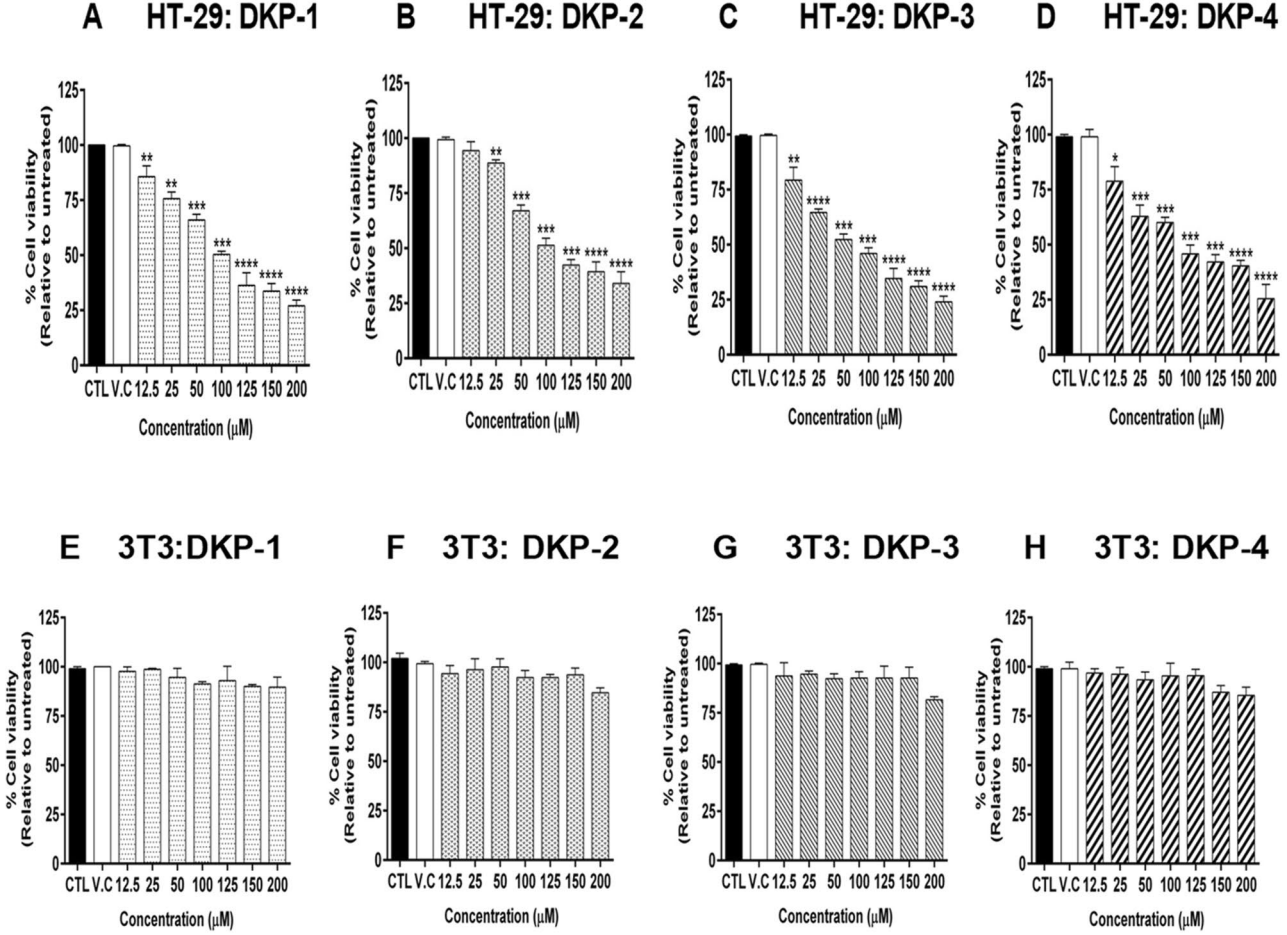
Cell viability in cells treated with cyclic dipeptides (DKPs). (**A**–**D**) Colorectal cancer HT-29 cells were treated with different doses of DKP-1: cyclo (l-Pro-l-Leu) (**A**), DKP-2: cyclo (l-Pro-l-Val) (**B**), DKP-3: cyclo (l-Pro-l-Phe) (**C**), and DKP-4: cyclo (l-Pro-l-Tyr) (**D**) for 24 h. (**E**–**H**) Normal mouse fibroblast 3T3 cells were treated with different doses of DKP-1 (**E**), DKP-2 (**F**), DKP-3 (**G**), and DKP-4 (**H**) for 24 h. Cell viability was determined relative to the untreated cells or vehicle control. Data were expressed as mean ± SD (n = 3). Data were analyzed by one-way ANOVA with student’s two-tailed *t*-test. The asterisks *p < 0.05, **p < 0.001, ***p < 0.0001, ****p < 0.0001, indicates a significant difference between the control in response to DKPs treaments. *CTL* Control; *VC* Vehicle control (DMSO).

MTT assay revealed that the four DKPs affected the viability of HT-29 cells in a dose-dependent manner when compared with untreated or DMSO treated cells (Fig. 2). After 24 h treatment with a 125 μM dose of four DKPs, the relative percentage viability of HT-29 cells was 36.33 ± 5.08% (DKP-1), 42.33 ± 2.25% (DKP-2), 34.66 ± 4.03% (DKP-3), and 42.15 ± 2.94% (DKP-4) (Fig. 2A–D). Colorectal cancer HT-29 cells inhibitory (IC_50_) doses were for 100.5 μM (DKP-1), 117.70 μM (DKP-2), 85.19 μM (DKP-3), and 97.97 μM (DKP-4). Mouse embryonic fibroblast (3T3) cells were used as control cells to compare the cytotoxic effects of DKPs in tumor and normal cells. The results showed that all DKPs did not exhibit a significant cytotoxic effect in 3T3 cells, while we observed the viability loss of > 10% in the higher dose of 200 μM treated cells (Fig. 2E–H). Moreover, these four DKPs were exhibited promising antiproliferative activity against HT-29 cells in this study.

### Induction of apoptosis by DKPs in HT-29 cells

**Figure 3:**
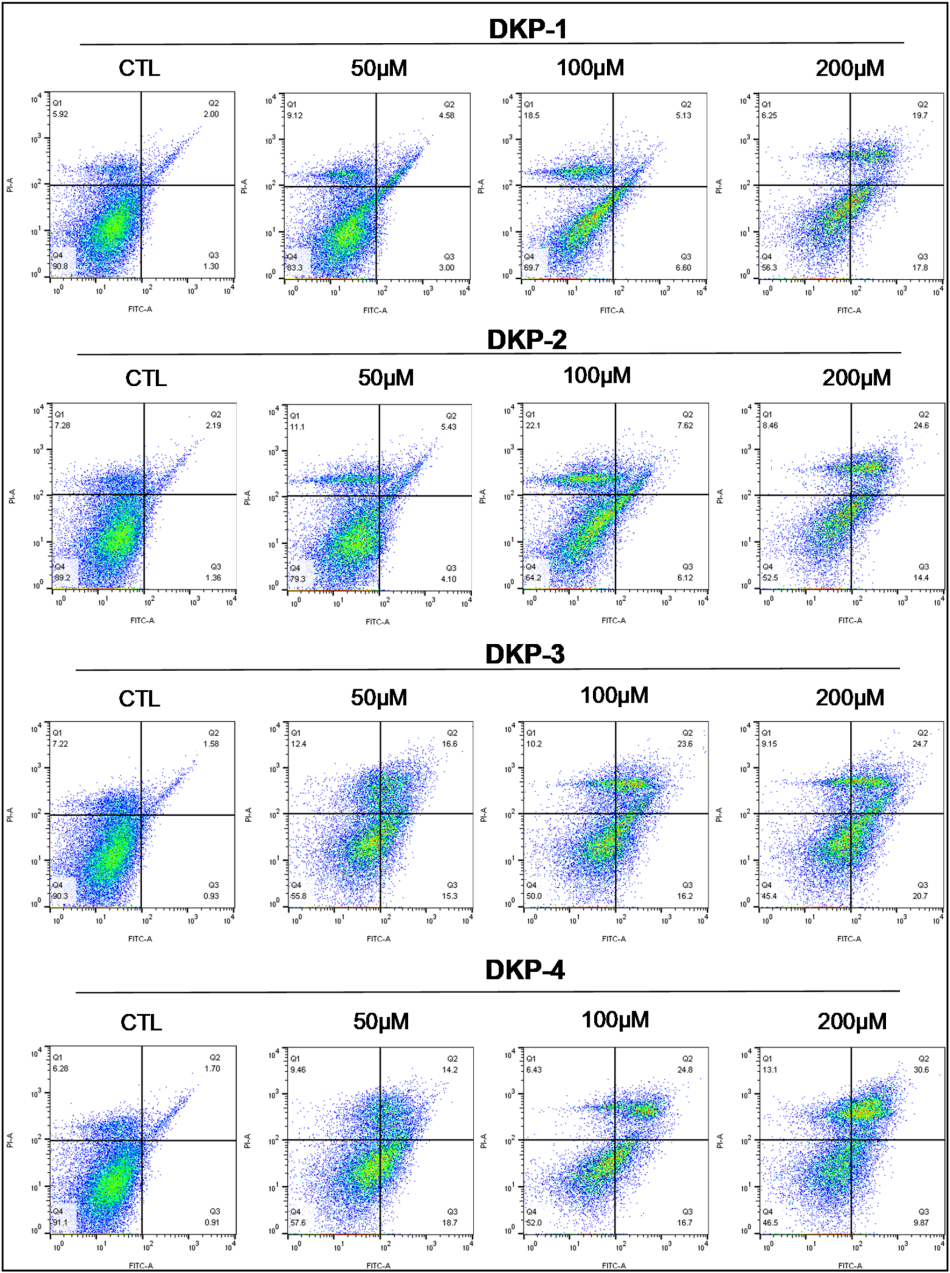
Cyclic dipeptide induces apoptosis in HT-29 cells. Annexin V-FITC/PI double staining was performed to analyze apoptotic cell populations by flow cytometry. HT-29 cells were treated with different doses (50, 100 and 200 μM) of four DKPs as indicated for 24 h. Percentage of the dead (Q1), late apoptotic/necrotic (Q2), early apoptotic (Q3) and viable (Q4) cells were shown in quadrant plot. *CTL* Control.

To determine whether the growth inhibitory effects of DKPs were linked to the induction of apoptosis by annexin V-FITC and propidium iodide staining was evaluated. We found that treatment of HT-29 cells with DKPs for 24 h increased the number of early (PI^*–*^/AV^+^; Q3) and late apoptotic or necrotic (PI^+^/AV^+^; Q2) cells in a dose-dependent manner (Fig. 3 and Table 2). Also, we observed that the populations of dead cells (PI^+^/AV^*–*^; Q1) have changed, 3.1-fold (DKP-1), 3.0-fold (DKP-2), 1.7-fold (DKP-3), and 2.1-fold (DKP-4) at a dose of 100 μM DKP-1–2, 50 μM DKP-3, and 200 μM DKP-4 treated cells when compared with untreated cells (Fig. 3 and Table 2). Moreover, both dead (Q1 + Q2) and apoptotic (Q2 + Q3) cell populations were significantly higher in the DKPs treatments compared to the control (Table 2). However, apoptotic cells were differentiated from necrotic cells by co-staining with PI because PI stains necrotic cells and it was excluded from live and early apoptotic cells due to the presence of an intact plasma membrane. This result revealed that DKPs inhibits the growth of HT-29 cells by the induction of apoptosis.

**Table 2:** Cyclic dipeptides induces apoptosis in HT-29 cells in a dose-dependent manner.

Concentration in μM	Q1	EAC Q3	LA/NC Q2	Q1 + Q2	Q2 + Q3
**Fold changes**					
DKP-1: HT-29					
CTL	1.0	1.0	1.0	1.0	1.0
50	1.5	0.4	2.3	1.7	2.3
100	3.1	0.9	2.6	3.0	3.6
200	1.1	2.4	9.9	3.3	11.4
DKP-2: HT-29					
CTL	1.0	1.0	1.0	1.0	1.0
50	1.5	3.0	2.5	1.7	2.7
100	3.0	4.5	3.5	3.1	3.9
200	1.2	10.6	11.2	3.5	11.0
DKP-3: HT-29					
CTL	1.0	1.0	1.0	1.0	1.0
50	1.7	16.5	10.5	3.3	12.7
100	1.4	17.4	14.9	3.8	15.9
200	1.3	22.3	15.6	3.8	18.1
DKP-4: HT-29					
CTL	1.0	1.0	1.0	1.0	1.0
50	1.5	20.5	8.4	3.0	12.6
100	1.0	18.4	14.6	3.9	15.9
200	2.1	10.8	18.0	5.5	15.5

Annexin V-FITC/PI double staining was performed to analyze apoptotic cell populations. HT-29 cells were treated with different doses of four DKPs as an indicated time for 24 h. Data are representative of three independent experiments (n = 3). DKP -1—cyclo (l-Pro-l-Leu), DKP -2—cyclo (l-Pro-l-Val), DKP -3—cyclo (l-Pro-l-Phe), and DKP -4—cyclo (l-Pro-l-Tyr). *CTL* Control, Dead cells (Q1), EAC—early apoptotic cells (Q3), LA/NC—late apoptotic or/and necrotic cells (Q2), Q1 + Q2— total dead cells, Q2 + Q3—total apoptotic cells.

### Effects of DKPs on the expression of apoptosis-related genes

**Figure 4:**
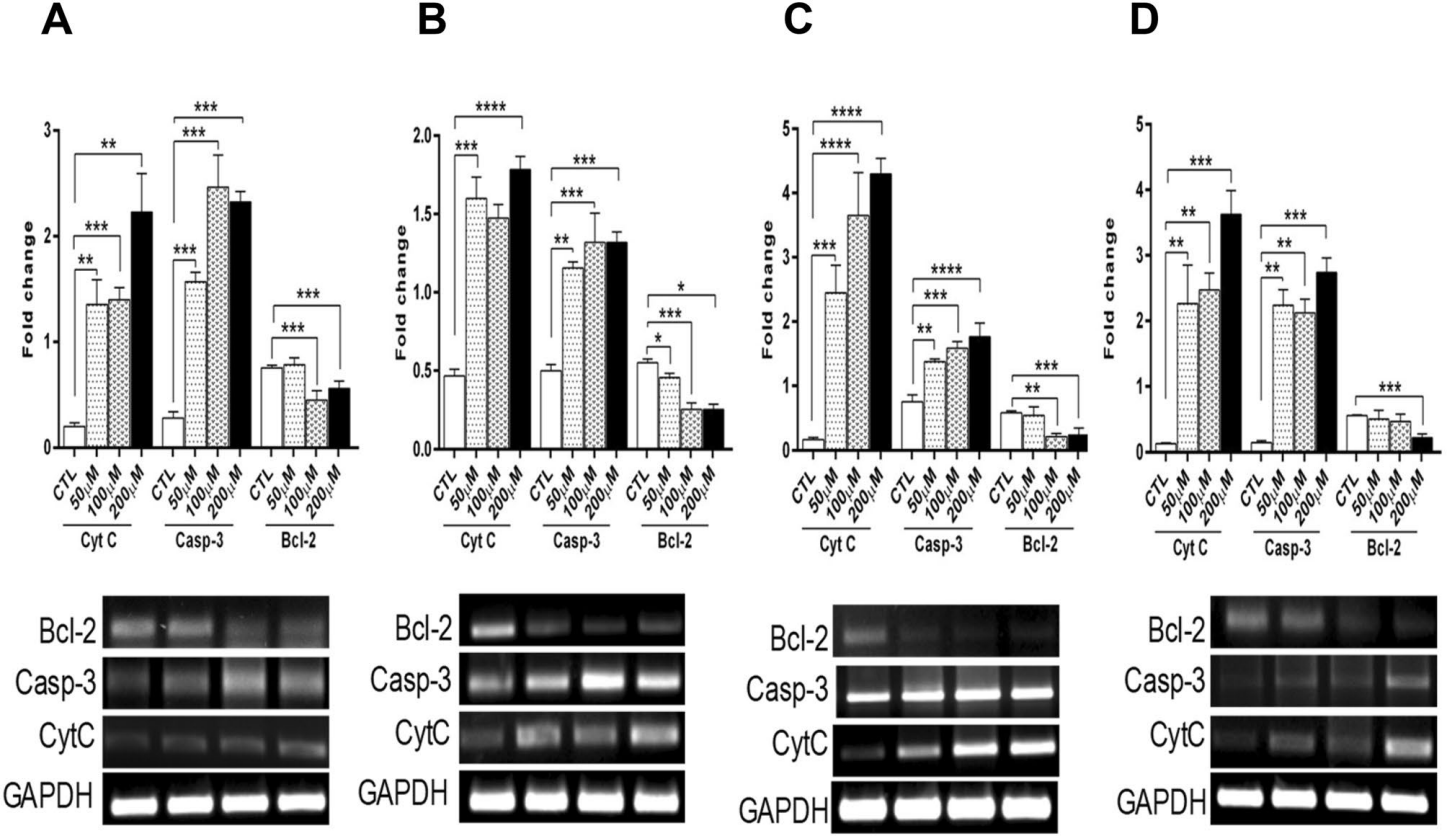
Apoptotic and anti-apoptotic gene expression levels in DKPs treated and un-treated HT-29 cells for 24 h. DKP-1: cyclo (l-Pro-l-Leu) (**A**), DKP-2: cyclo (l-Pro-l-Val) (**B**), DKP-3: cyclo (l-Pro-l-Phe) (**C**), and DKP-4: cyclo (l-Pro-l-Tyr) (**D**). The sqRT-PCR analysis was performed in triplicates, and each bar represents the mean ± SD (n = 3). Data were analyzed by one-way ANOVA with student’s two-tailed *t*-test. The asterisks *p < 0.05, **p < 0.001, ***p < 0.0001, ****p < 0.0001, indicates a significant difference between the untreated cells in response to DKPs treated cells. The groupings were cropped from different gels subjected to identical conditions. Full gel images were shown in the Supplementary Information, Fig. S3A–D.

The expression levels of Cytc and Casp-3 were significantly (*p* < 0.001) up-regulated in a dose-dependent manner in response to DKPs treatments compared to the control (untreated cells) (Fig. 4). Cytc levels were significantly up-regulated, 2.32-fold, 1.78-fold, 4.29-fold, and 3.62-fold, respectively, in DKP-1, DKP-2, DKP-3 and DKP-4 at a dose of 200 μM compared with untreated cells (*p* = 0.001; Fig. 4A–D). Similarly, Casp-3 gene expression was up-regulated, 2.32-fold (DKP-1), 1.31-fold (DKP-2), 1.76-fold (DKP-3), and 2.73-fold (DKP-4) at a dose of 200 μM DKPs treated cells compared with untreated cells (*p* = 0.0001; Fig. 4A–D). Interestingly, the anti-apoptotic gene (Bcl-2) expression level was significantly down-regulated in HT-29 cells in response to DKPs treatments compared to the control. Moreover, the induction of apoptosis by DKPs was linked with the suppressed mRNA expression level of Bcl-2 in HT-29 cells (Fig. 4A–D).

### Effect of DKPs on the expression of apoptotic proteins

**Figure 5:**
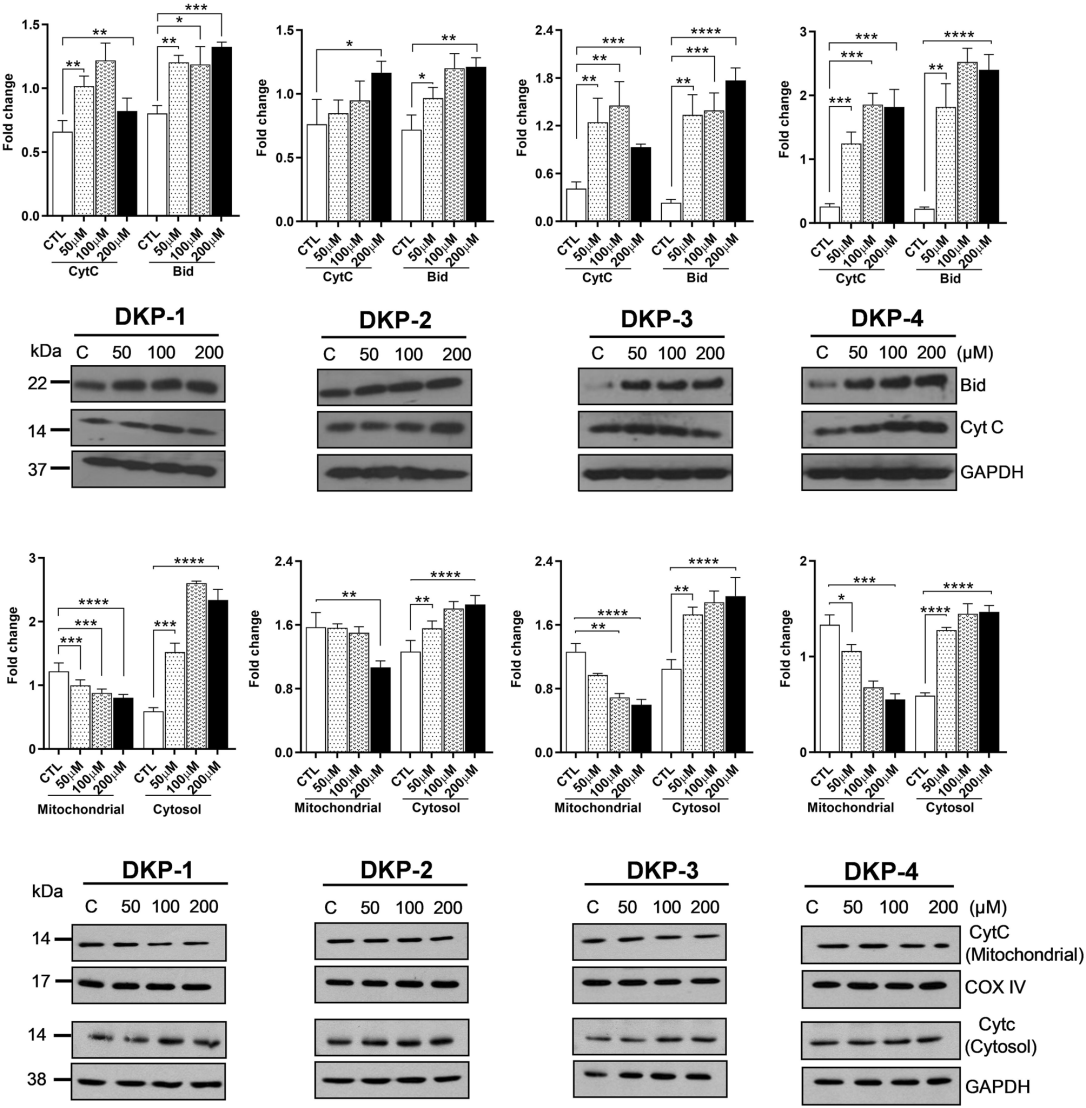
Apoptotic protein expression levels in DKPs treated and un-treated HT-29 cells for 24 h. (**A**–**D**) Effect of DKPs on the expression of apoptosis-related proteins in the whole-cell lysate of DKP-1: cyclo (l-Pro-l-Leu) (**A**), DKP-2: cyclo (l-Pro-l-Val) (**B**), DKP-3: cyclo (l-Pro-l-Phe) (**C**), and DKP-4: cyclo (l-Pro-l-Tyr) (**D**) for 24 h. (**E–H**) Effect of DKPs on the cytochrome-c levels from the mitochondrial and cytosolic fraction of DKP-1: cyclo (l-Pro-l-Leu) (**E**), DKP-2: cyclo (l-Pro-l-Val) (**F**), DKP-3: cyclo (l-Pro-l-Phe) (**G**), and DKP-4: cyclo (l-Pro-l-Tyr) (**H**). Western blot analysis was performed using antibodies against CytC, Bid, and GADH served as a housing gene. Data were presented as the mean ± SD (n = 3). Data were analyzed by one-way ANOVA with student’s two-tailed *t*-test. The asterisks *p < 0.05, **p < 0.001, ***p < 0.0001, ****p < 0.0001, indicates a significant difference between the control in response to DKPs treaments. The groupings were cropped from different gels subjected to identical conditions. Full blots were shown in the Supplementary Information, Fig. S4A–H.

**Figure 6:**
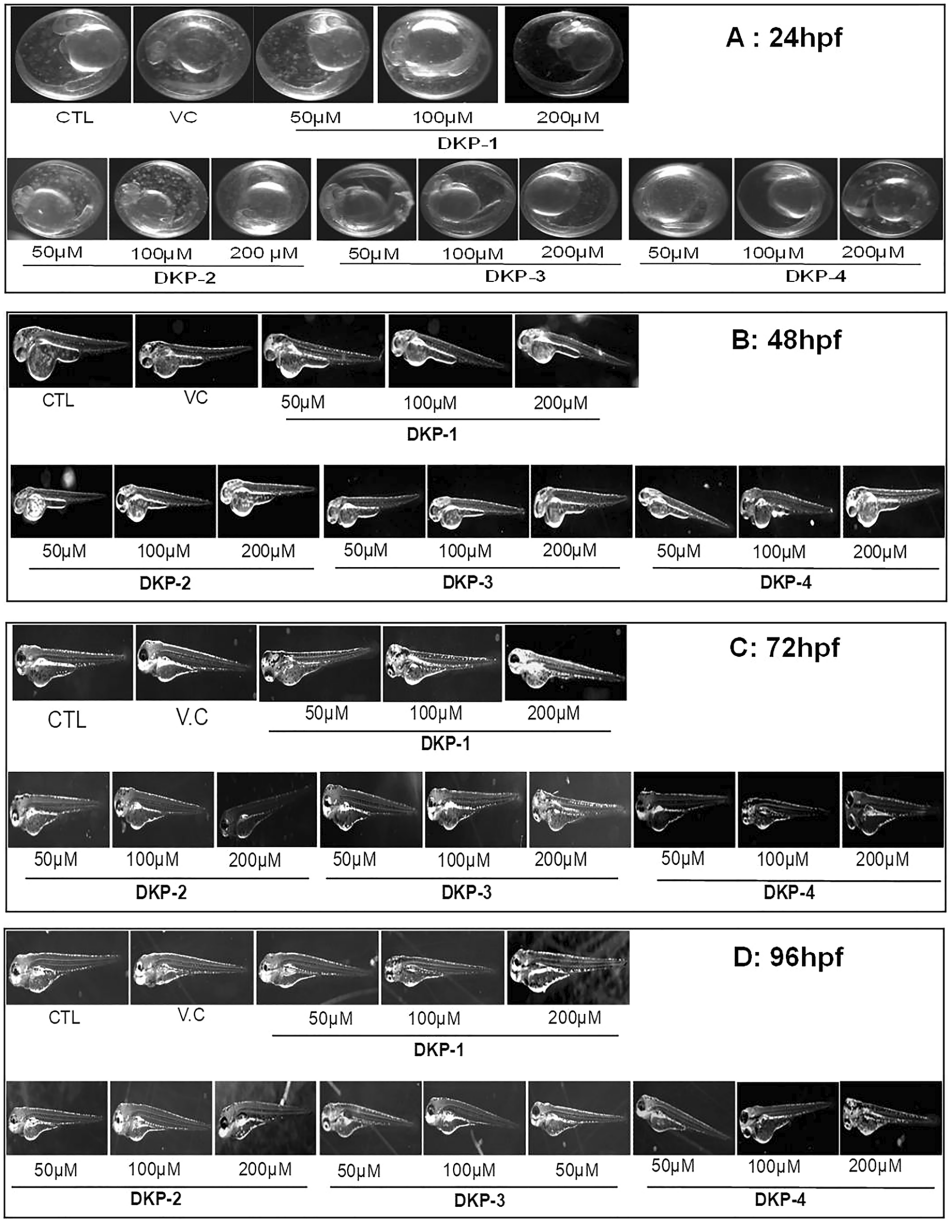
Developmental changes in the zebrafish embryos after treatment with different doses of four cyclic dipeptides. Representative images for 24 hpf (**A**), 48 hpf (**B**), 72 hpf (**C**), and 96 hpf (**D**). *CTL* Control, *DMSO* Vehicle control (DMSO).

The expression of apoptosis-related proteins (Cytc and Bid) following DKPs treatment were evaluated by Western blot analysis to further determine the mechanism of DKPs induced apoptosis. We observed that all DKPs had a significant increase in the expression levels of apoptotic proteins compared with untreated cells (Fig. 5). Cytc expression was significantly up-regulated, 1.21-fold (DKP-1), 1.45-fold (DKP-3), and 1.85-fold (DKP-4), at a dose of 100 μM of DKPs treatment compared with untreated cells (*p* = 0.0040, *p* = 0.0045, and *p* = 0.0001, respectively; Fig. 6A–D). In contrast, the expression levels were slightly decreased at a dose of 200 μM treated cells compared to lower doses of 50 μM and 100 μM respectively (Fig. 5A,C,D). Likewise, the Bid expression was up-regulated, 1.32-fold 1.21-fold 1.76-fold, respectively in DKP-1, DKP-2 and DKP-3 at a treatment dose of 200 μM compared to the untreated group (*P* < 0.001) (Fig. 5A–D). Interestingly, we found that DKP-4 treatment with a lower dose of 100 μM increased the expression level of Bid when compared with control and the higher dose of 200 μM treated HT-29 cells (Fig. 5C). Further, the expression level of Cytc was increased in the cytosolic fraction in response to DKPs treatments, whereas the level of Cytc decreased in the mitochondrial fraction (Fig. 5E,H) when compared to the untreated cells. Moreover, this result indicated that Cytc was released from mitochondria into the cytosol in a concentration-dependent manner and also triggered the mitochondria-mediated apoptotic cascade.

### Effects of DKPs on zebrafish embryo developmental toxicity

Zebrafish (ABiC) embryos were treated with different doses of four DKPs for 96 hpf. At every 24 h time interval, the survival rate and developmental changes of the embryos were observed by inverted fluorescence microscope. We observed no significant decrease in the survival rate of embryos treated with various doses (50, 100 and 150 μM) of three DKPs (DKP-1, DKP-2 and DKP-4) treaments compared with untreated embryos as well as DMSO treated embryos at 96 hpf. Also, the survival rates of embryos were slightly higher in the treatment groups compared to the control (Table 3). The survival rate was higher at lower doses (50 and 150 μM) of DKP-3 treated embryos, whereas the survival rate was slightly reduced at the higher dose of 200 μM treated embroys compared with untreated embryos (Table 3). Moreover, we confirmed the morphological features of the zebrafish embryos after treatment with four DKPs using an inverted microscope*.* As evident from the images, all the DKPs did not show any adverse effects on the developmental toxicity or teratogenic effect on the embryos at 24, 48, and 72 hpf exposure (Fig. 6). Furthermore, beyond 96 hpf exposures at a higher dose of 200 μM, all DKPs, none of the DKPs showed any side effects on the embryonic development of the zebrafish (Fig. 6A–D).

**Table 3:** Mean survival rates (in three replicates) of DKPs treated and untreated zebrafish embryos.

Concentration in μM	DKP-1		DKP-2		DKP-3	DKP-4	
**24 hpf**							
CTL	73.33 ± 0		73.33 ± 0		73.33 ± 0	73.33 ± 0	
50	70.88 ± 3.44		77.77 ± 5.41		80 ± 5.96	79 ± 3.96	
100	73.33 ± 5.96		82.22 ± 4.41		80 ± 5.96	82 ± 2.96	
200	75 ± 5.77		75.55 ± 3.44		75.55 ± 4.12	71.55 ± 5.12	
**48 hpf**							
CTL	71.11 ± 3.44		71.11 ± 3.44		71.11 ± 3.44	68.88 ± 2.66	
50	70.88 ± 5.96		80 ± 5.96		80 ± 5.96	79 ± 3.96	
100	71.11 ± 1.54		80 ± 5.96		80 ± 5.96	81 ± 2.96	
200	73.33 ± 3.32		75.55 ± 3.44		75.55 ± 4.12	70.34 ± 3.33	
**72 hpf**							
CTL	68.88 ± 3.44		68.88 ± 3.44		68.88 ± 3.44	68.88 ± 3.44	
50	71.11 ± 3.44		68.88 ± 3.44		77.77 ± 9.10	75.51 ± 3.32	
100	71.11 ± 5.55		77.33 ± 3.44		80 ± 5.96	79.3 ± 2 .77	
200	68.88 ± 6.91		71.11 ± 6.88		73.33 ± 5.96	70.54 ± 3.51	
**96 hpf**							
CTL	68.88 ± 3.44		66.66 ± 3.44		68.88 ± 3.44	68.88 ± 3.44	
50	66.66 ± 5.96		66.66 ± 5.96		68.88 ± 6.88	71.11 ± 2.44	
100	71.11 ± 9.10		71.11 ± 9.10		80 ± 5.96	79.3 ± 2.77	
200	66.66 ± 5.96		66.66 ± 5.96		69.22 ± 12	68.24 ± 5.89	

Data are represented as mean ± SD (n = 3). DKP-1: cyclo (l-Pro-l-Leu) (**A**), DKP-2: cyclo (l-Pro-l-Val) (**B**), DKP-3: cyclo (l-Pro-l-Phe) (**C**), and DKP-4: cyclo (l-Pro-l-Tyr) (**D**). hpf – hours post fertilization; *CTL* Control.

### Anti-cancer effects of dipeptides in a xenograft model

**Figure 7:**
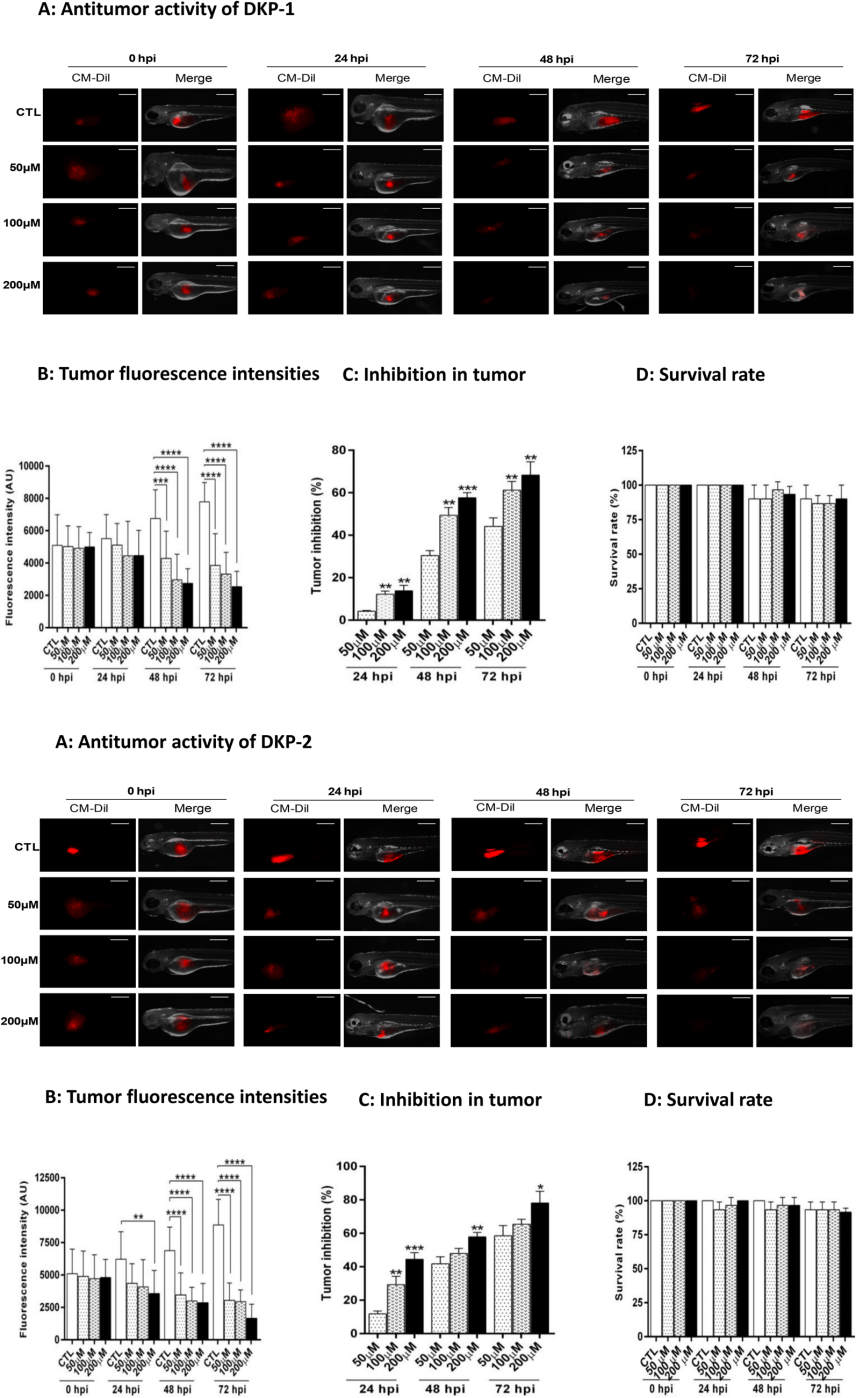

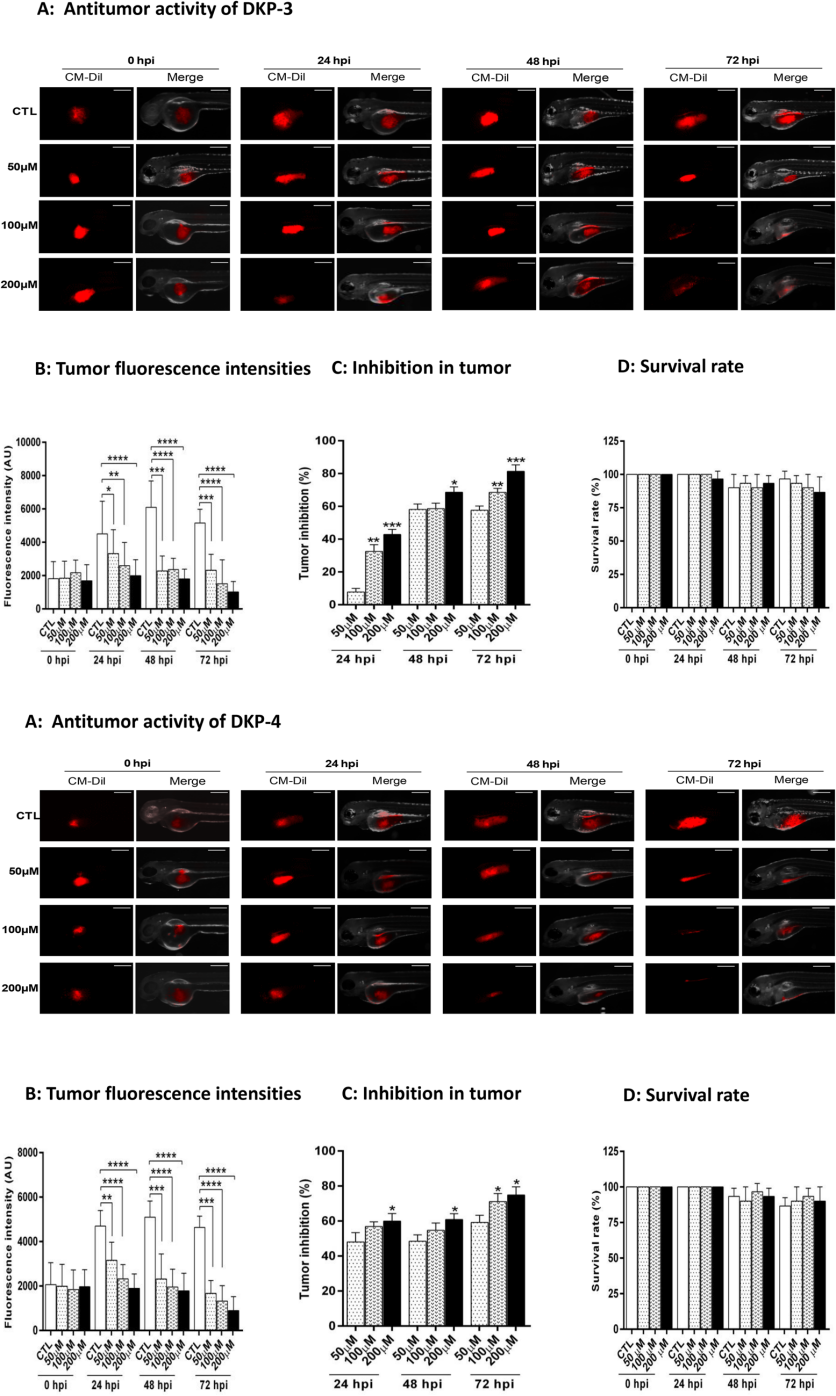
Antitumor effects of four cyclic dipeptides in a zebrafish xenograft model. On 2 dpf, CM-Dil labelled HT-29 xenografted embryos in DKPs treated and un-treated larvae for 72 h. Representative fluorescent images showing DKPs treated and un-treated HT-29 xenograft zebrafish larvae at 0 hpi, 24 hpi, 48 hpi and 72 hpi respectively (**A**). Quantification of the fluorescence intensity of tumor (**B**). Tumor growth inhibition rates were calculated according to the fluorescence intensities of the tumor (**C**). Bar graph showing the survival rate of DKPs treated and un-treated xenografted zebrafish larvae (**D**). Data were analyzed by one-way ANOVA with student’s two-tailed *t*-test. The asterisks *p < 0.05, **p < 0.001, ***p < 0.0001, ****p < 0.0001, indicates a significant difference between the control in response to DKPs treatments. *CTL* Control; Scale bar = 200 µm. The groupings were cropped from different zebrafish/tumor xenograft images subjected to identical conditions. Full zebrafish xenograf embroys were shown in the Supplementary Information, Fig. S5A-D. *CTL* Control.

The antitumor effects of all DKPs were further investigated in a zebrafish xenograft model. We observed a significant (*p* < 0.001) reduction in the fluorescence intensity and inhibition of tumor growth (> 30%) by DKP-2, DKP-3, and DKP-4 compared with control and treatment with DKP-1, after 24 hpi (Fig. 7 and Fig. S5). After 48 hpi, the inhibition of tumor growth rate was significantly increased, 57% (DKP-1), 58% (DKP-2), 68% (DKP-3), and 60% (DKP-4), in the dose of 200 μM treated cells compared to the untreated cells (*p* = 0.0001, *p* = 0.0049, *p* = 0.0174, and *p* = 0.0123, respectively; Figs. 7 and S5). Moreover, tumor growth was significantly reduced at the dose of 200 μM treated embroys; DKP-1 (68%), DKP-2 (78%), DKP-3 (81%) and DKP-4 (74%) compared with control embroys at 72 hpi. At all time intervals, zebrafish were treated with different doses of DKP-3 exhibited the highest tumor growth inhibition rates when compared to other peptides used in this study (Figs. 7; S5). Furthermore, the survival rate was slightly reduced in response to DKPs treatment compared to the control, after 48 hpi and 72 hpi (Figs. 7; S5).

## Discussion

Several investigations demonstrated the reduced risk of developing CRC in humans consuming diets rich in probiotics^26^. The principle probiotics derived agents such as cyclic peptides, conjugated linoleic acids (CLAs) and short-chain fatty acids (SCFAs) could protect against cancer and infectious diseases^26,27^. Among them, DKPs were demonstrated to exert their effects on various biological functions including inhibiting the cell cycle, inducing apoptosis, reducing oxidative stress, and stimulating the immune responses^5,26^. In this study, the viability assay revealed that four DKPs induced growth arrest in HT-29 cells in a dose-dependent manner. Subtle structural variances are known to play a vital role in the inhibition of all DKPs. Besides, the tested four DKPs did not exhibit a cytotoxic effect on normal 3T3 cells compared to the untreated cells. The growth-inhibitory effects of DKPs are similar to the previous report on similar groups of compounds, where cyclo (Phe-Pro) and cyclo (Tyr-Pro) exhibited 50% growth inhibition of HT-29, HeLa, and MCF-7 cells in a dose-dependent manner after treatment with a dose of fewer than 7 mM^12^. The crude mixture of DKPs obtained from *P. aeruginosa* PAO1 strain, mainly comprised of cyclo (l-Pro-l-Val), cyclo (l-Pro-l-Try), and cyclo (l-Pro-l-Phe), induced cell death in cervical and colon cancer cells with IC_50_ values of 0.53 and 0.66 mg/mL, respectively^13^. A new cyclic dipeptide petrocidin-A was isolated from *Streptomyces* sp. SBT348, which exhibited significant anti-proliferative effects towards HL-60 and HT-29 cells with IC_50_ values of 3.9 and 5.3 μg/mL^28,29^. Moreover, cyclo (l-Leu-l-Pro) exhibited anticancer effects against three leukemic cell lines at a concentration of less than 100 µg/mL^30^. A possible explanation for the low and high peptide concentrations required for significant growth inhibition of various cancer cells described above is owing to several factors including the possibility of DKPs binding to serum proteins in the culture medium, and drug efflux by P-glycoprotein, or the lack of sufficient absorption of peptides into the cells^25^.

A key mechanism involved in the action of many anticancer drugs is the activation of extrinsic and intrinsic apoptotic pathways^31–34^. The crucial element in the intrinsic apoptotic pathway is the efflux of Cytc from mitochondria to the cytosol. In the cytosol, Cytc together with apoptotic protease activating factor 1 (Apaf-1) activates Casp-9, which activates Casp-3 as a key execution of apoptosis. In both pathways, the initiator caspase cleaves and activates downstream effector caspase, such as Casp-3^35^. In this study, we noted that all DKPs induce early and late apoptotic cell death of HT-29 cells in a dose-dependent manner. Taken together, both MTT and apoptosis analysis revealed that four DKPs are robustly inhibiting the proliferation of colon adenocarcinoma cells by the induction of apoptosis. Further, we observed the expression levels of apoptotic (Cytc, Casp-3 and Bid) and anti-apoptotic (Bcl-2) markers were up- and down-regulated in HT-29 cells in response to DKPs treatments. A possible explanation for the significant up-regulation of Casp-3 in DKPs treated cells, in this study may be activated by an initiator caspase such as Casp-9^36,37^. However, all DKPs induces apoptosis by directly activating Casp-3 or by cleaving Bid (BH3 interacting-domain death agonist), resulting in mitochondrial dysfunction and subsequent release of Cytc and activation of Casp-9 and Casp-3^17^. Our findings are supported by an earlier study, where the expression of apoptotic genes was significantly up-regulated in oral squamous cancer after treatment with isocudraxanthone K^36^. Lee and co-workers also reported that anthocyanin, the derivative of the flavonoid family, which induces apoptosis of leukaemia U937 cells by down-regulating Bcl-2 expression^37^. Our observations are consistent with previous studies suggesting that mitochondria play an important role in cell survival and mitochondrial-mediated apoptosis by modulating the balance of pro and anti-apoptotic Bcl-2 family proteins^38–40^. Besides, DKPs were isolated from *P. aeruginosa* PAO1, which induces apoptosis in caspase-9-dependent pathway^41^. Overall, our findings reveal that DKPs induces apoptosis in HT-29 cells by the activation of a mitochondria-mediated apoptotic pathway.

In recent years, zebrafish become a suitable model for oncology research using the approach of xenotransplantation of human cancer cells into the yolk sac of a zebrafish embryo. Nowadays, zebrafish embryos are widely used to study the toxicity assessment of various drugs and bioactive metabolites due to its experimental reliability and also low expansive model^42,43^. Our toxicity study revealed that four DKPs did not show any significant mortality rate and developmental changes of the zebrafish embryos after exposure with 96 hpf. Besides, the tested four DKPs had a promising antitumor effect on HT-29 xenograft zebrafish. In agreement with our findings, three anti-angiogenic drugs such as bevacizumab, endostar, and lapatinib were demonstrating a remarkable inhibition of tumor growth in a xenografted zebrafish model within a nonlethal dose range^44^. Fu and co-workers reported that pan-phosphatidylinositol 3-kinase (PI3K) inhibitor LY294002 significantly reduced the xenograft tumor size in zebrafish by decreasing the viability of metastatic cancer cells^45^. Furthermore, small-molecule bromodomain-containing protein-4 (BRD4) inhibitor displayed a potent antitumor effect on the zebrafish breast cancer model by targeting BRD4 without causing any cytotoxic effect^46^. To the best of our knowledge, we report for the first time, DKPs have an antitumor therapeutic potency as a lead drug of CRC in vivo zebrafish xenograft model.

In conclusion, four DKPs were isolated and identified from the ethyl acetate extract in the cell free-filtrate of *E. acetylicum* S01, for the first time in this study. These four DKPs were inhibited cell growth arrest and the induction of apoptosis through the activation of the mitochondria-mediated apoptotic pathway (Fig. S6). Moreover, in vivo results revealed that tested four DKPs significantly inhibited tumour progression in a zebrafish xenograft model. Overall, our findings suggest that cyclic dipeptides would be promising drug candidates for CRC therapeutics. This finding paves the path for future studies into the activation and regulation of the mitochondria-mediated apoptotic pathway in CRC by proline-rich cyclic peptides.

## Materials and methods

### Bacterial strain, extraction, and purification of bioactive compounds

Strain *E. acetylicum* S01 was genetically confirmed by 16S ribosomal RNA gene sequencing, and it showed desirable functional probiotic attributes and provided disease resistance against *Aeromonas hydrophila* infection in goldfish (*Carassius auratus*) as described earlier^47,48^. For identification and characterization of bioactive compound(s), a loopful culture of *E. acetylicum* S01 was grown in Luria–Bertani broth (LB) (casein enzymatic hydrolysate 10 g/L, yeast extract 5 g/L, sodium chloride 10 g/L, pH 7.5 ± 0.2, water 1,000 mL) and incubated on a rotary shaker (120 rpm) at 37 °C. When the absorbance of the culture was approximately 1.5 at 600 nm, the log phase culture was transferred aseptically into the 400 mL sterile medium and incubated in an orbital shaker at 37 °C for 72 h. The fermented cultures were then centrifuged (6,500 × g, 15 min at – 4 °C) followed by filtration through a 0.45-µm filter, to obtain the cell-free culture filtrate. The cell-free supernatants (25-L) obtained after cultivation of the strain S01 in a 1-L conical flask for 96 h were then extracted twice with an equal volume (1:1 ratio) of ethyl acetate and the organic layer was concentrated by rotary evaporator (IKA-RV10, Switzerland). After concentration, the crude extract was yielded 1.3 g/25 L of culture filtrate.

### Purification and structure elucidation of bioactive compounds

The oily reddish-yellow residue S01 (1.3 g) obtained after drying was then loaded on a silica gel column (30 cm × 2 cm) previously equilibrated with hexane and eluted consecutively using a linear gradient hexane/ethyl acetate (80:20, 70:30, 60:40, 50:50, 40:60, 30:70, v/v) followed by chloroform/methanol (9:3, v/v) was finally added into the column for elution. The ethyl acetate extract yielded four compounds of pale yellow crystalline nature. The structural elucidation of the isolated compounds was determined by nuclear magnetic resonance (NMR) spectroscopy (Bruker DRX-500-MHz, Rheinstetten, Germany) using denudated chloroform (CDCl_3_) as a solvent. ^1^H and ^13^C NMR spectra were recorded at ambient temperature at 500-MHz equipped with a 2.5-mm microprobe. Chemical shifts were referred to as CDCl_3_ (δH 7.26 and δC 77.0), which is given in parts per million (ppm) and coupling constant in Hertz. The compounds were analyzed by a high-resolution mass spectrophotometer (HRMS) and m/z values were obtained using the electrospray ionization mode (Orbitrap LC-Mas, Thermo Scientific Exactive, Waltham, USA) equipped with a BEH C-18 column (2.1 × 50 mm, Waters, Milford, USA). The specific optical rotation of the compounds was measured using a Jasco P-2000 digital polarimeter coupled with a sodium lamp (Na) at a wavelength of λ589 nm. Specific optical rotation was determined by using the following formula:

$$[{\mathrm{\alpha }]}_{\mathrm{D}}^{\mathrm{t}}= \frac{\mathrm{\alpha }}{\mathrm{cl}}$$

where *α* = observed rotation, *c* = concentration (g/mL), *l* = length of cell (dm), *D* = yellow of light from sodium lamp, and *t* = temperature (Celsius).

### Cell culture

Human colorectal cancer (HT-29) and mouse embryonic fibroblast (3T3) cells were obtained from the American Type Culture Collection Centre (ATCC, VA, USA). Both cells were grown in Dulbecco Modified Eagle's Medium (DMEM, HiMedia, India) supplemented with 10% heat-inactivated fetal bovine serum (FBS), penicillin (50 units/mL), and streptomycin (50 µg/mL) and were cultured at 37 °C, 5% CO_2_ in a humidified atmosphere.

### Cell viability by the MTT assay

Cytotoxicity of the identified those DKPs were determined by cell viability study with the MTT (3-(4, 5-dimethylthiazol-2yl)-2, 5-diphenyltetrazolium bromide) reduction assay^﻿49^. Briefly, 1.5 × 10^4^ cells/well (HT-29) and 1.7 × 10^4^ cells/well (3T3) cells were seeded into 96-well plates. They were incubated for 24 h at 37 °C with 5% CO_2_ and 95% relative humidity atmosphere. After 24 h, identified DKPs were added different concentrations (12.5 to 200 µM) into culture media in triplicates and re-incubated under the same culture conditions mentioned above. The final concentration of DMSO solution was 0.25% in the cells served as a negative control. Then culture media was removed from the plates, and 100 μL of freshly prepared MTT solution in serum-free medium (5 mg/mL) was added to each well and further incubated for 4 h. After that, 150 μL of DMSO solution was added under dark conditions and kept for 30 min at room temperature. The absorbance of the formazan product was measured at 595 nm in the microplate reader (Imark, Biorad). The percentage viability of the cells was measured by using the formula: Cell viability (%) = [(test/control) × 100)].

### Apoptosis analysis by Annexin-V/PI staining

The dead, necrotic, and apoptotic cells were analyzed by annexin V/PI double staining method, which was used to detect the externalization of phosphatidylserine (PS). Briefly, cells at the density of 4.5 × 10^4^ cells/well were seeded into 6-well plates and treated with or without DKPs for 24 h. Subsequently, both floating and adherent cells were harvested by centrifugation at 1,500 rpm for 5 min, then re-suspended in 300 μL of 1X binding buffer, followed by 3 μL of annexin V-FITC and propidium iodide (PI) (Apoptosis Detection kit, Lead Gen, Taiwan) was added into each sample and incubated for 20 min at room temperature in the dark following the protocol recommended by the manufactures. Cells were analyzed by flow cytometry (FACS Calibur, Becton Dickinson, CA, USA) using WinMDI 2.9 software (La Jolla, CA, USA). The individual populations can be defined using quadrant gates, the number of cells in each quadrant indicated the following: Quadrant-1 (Q1): annexin V^-^/PI^+^ cells were considered as dead cells; Quadrant-2 (Q2): Annexin V^+^/PI^+^ cells were considered as late apoptotic and necrotic cells; Quadrant-3 (Q3): Annexin V^+^/PI^−^ cells were considered as early apoptotic cells; Quadrant-4 (Q4): Annexin V^-^/PI^-^ cells were considered as healthy. Data were analyzed using FlowJo V12.1 software (Tree stat, Stanford, CA, USA).

### RNA isolation and gene expression analysis

Total RNA was isolated from HT-29 cells using the TRIzol reagent (TaKara, Japan) following the manufacturer’s instruction. The quantity and quality of each RNA sample were analyzed with a NanoDrop-1000 (Thermo Fisher Scientific, USA), followed by conversion to cDNA using RevertAid cDNA synthesis kit (Thermo Fisher Scientific, CA, USA) using 1 μg of mRNA to synthesize cDNA. Semi-quantitative reverse transcriptase-polymerase chain (sqRT-PCR) reaction was carried out in a Veriti Thermal Cycler (Applied Biosystems, CA, USA) using 2X Taq DNA polymerase Master Mix RED (Ampliqon, Denmark, Germany) as we previously described method^48^. Primer sequences for sq-RT-PCR are listed in Table 4. All sqRT-PCRs were run for at least three times. GAPDH served as a housekeeping gene to normalize the expression levels. The expression levels were analyzed by measuring the intensity of the band from the gel using Image-J software.

**Table 4:** List of primers used for sqRT-PCR amplifications.

Gene	Amplicon size	Anneal	Primer (5′–3′)
Bcl-2	267 bp	55 °C—35 s	FWD: CTTTGAGTTCGGTGGGGTCA
			REV: TGGTGATGTGAGTCTGGGCT
Casp-3	275 bp	59 °C—40 s	FWD: GCAGCAAACCTCAGGGAAAC
			REV: TGTCGGCATACTGTTTCAGCA
Cytc	302 bp	55 °C—40 s	FWD: AAAAGAGGTGAGAGCGGGTC
			REV: TGGTTGCTGAAGGGCTATGG
GAPDH	576 bp	59 °C—40 s	FWD: CCATCACCATCTTCCAGGAG
			REV: CCTGCTTCACCACCTTCTTG

*Bcl-2* B-cell lymphoma 2, *Casp-3* Caspase-3, *Cytc* Cytochrome-c, *GAPDH* Glyceraldehyde 3-phosphate dehydrogenase.

### Western blot analysis

Total protein was isolated from HT-29 cells by using lysis buffer. Cells were collected by centrifugation at 1,500 rpm for 5 min. After that, cell pellets were re-suspended with RIPA lysis buffer consisting of 1 mM protease inhibitor cocktail (PIC; Sigma-Aldrich; P8340) and lysed at – 4 °C for 1 h. After incubation, the protein was obtained by centrifugation at 13,000 × *g* for 15 min and kept for − 80 °C until analysis. Also, mitochondrial and cytosolic fractions were obtained by using fraction lysis buffer as described previously^50^ with minor modifications. Lysed cells were kept in ice for 10 min and then cells were triturated by a 27 gauge syringe (25 strokes) to become homogeneous followed by centrifugation at 13,000 × *g* for 30 min. The collected supernatant was the cytosolic fraction. After the pellet was washed with lysis buffer and it was dissolved in lysis buffer for protein quantification. Protein concentration was measured by Pierce BCA protein assay kit (Thermo Scientific Laboratories, Rockford, USA) according to the manufacturer instructions. Equal amounts of proteins from all samples were separated by 10% SDS–polyacrylamide gel electrophoresis (SDS-PAGE) and electrotransferred. Polyvinylidene difluoride (PVDF) membrane was blocked with 5% skimmed milk for 1 h, followed by incubation with primary (Cytochrome-c: sc-13560; Bid: sc-11423; COX-IV: #4850; 1:2000; GAPDH: #3683; 1:10,000) and HRP-conjugated secondary (Got Anti-mouse IgG: 20102; Got anti-rabbit IgG: 20202: 1:10,000) antibodies against specific proteins. The protein signals were detected by Enhanced Chemiluminescence (ECL) detection kit (Amersham Piscataway, NJ, USA). The protein expression levels were analyzed by measuring the intensity of the band from scanned films using Image-J software. GAPDH and COX-IV served as a housekeeping protein to normalize the expression levels.

### Zebrafish husbandry and toxicity assay

Laboratory animal care principles were followed and experimental procedures were conducted following guidelines established by the Institutional Animal Care, and Use Committee (IACUC), and every effort was made to minimize suffering. This study was approved by the IACUC of the Kaohsiung Medical University, Kaohsiung, Taiwan (Approval No: KMU-IACUC-105213). The wild-type (ABiC strain) zebrafish (*Danio rerio*) were maintained at 28 °C on a 14 h/10 h light/dark cycle under standard circulating water systems and fed with hatched brine shrimp. Embryos were obtained by natural mating male fish with female fish (2:3) in a water tank and eggs were maintained in a plastic Petri dish containing ad libitum water. After 4 h incubation, embryos were randomly divided into five experimental groups (control (without treatment), sham/vehicle control (DMSO), and three treatment groups for each cyclic dipeptide. Each group had 45 embryos per test dosage. All DKPs were tested at different doses varied from 50, 100 and 200 μM, respectively. After drug treatment, embryos were maintained in an incubator at 28 °C. The survival rate and embryonic developmental changes were observed by using an inverted microscope every 24 h interval for 96 h (Nikon Eclipse TE2000-U, Tokyo, Jap).

### Zebrafish and tumor xenograft model

For cell labelling, HT-29 cells were incubated with cell tracker CM-Dil (Invitrogen, Carlsbad, CA, USA) dye at a final volume was 20 µL/mL for 25 min at 37 °C. To remove the unstrained dye, cells were washed twice and re-suspended with 1X PBS, pH 7.4 to a final density of 2.1 × 10^6^ cells/mL. Prior transplantation, CM-Dil labelled cells were assessed for viability using trypan blue exclusion assay. More than > 90% of viable cells were used. For xenotransplantation, 48-hours post-fertilization (hpf) wild-type ABiC embryos were anaesthetized in 0.01% tricaine methanesulfonate (Sigma-Aldrich; MS222) solution containing 0.3% phenylthiocarbamide (PTU; Sigma-Aldrich; P7629). After anesthetization, CM-Dil (a lipophilic fluorescent tracking dye) labelled HT-29 cells grafted into the yolk sac of each zebrafish embryos by using a microinjector IM-300 (Narishige, Tokyo, Japan). After injection, embryos were incubated for 1 h at 28 °C. For confirmation of the visible cell mass at the injection site, zebrafish were transferred to an incubator and maintained at 37 °C.

### Xenograft model for antitumor assay

Five hundred CM-Dil labelled HT-29 xenograft zebrafish embryos were randomly selected and hosed into 4 replicate wells in 12-well cell culture plates (5 embryos/well; 20 embryos/treatment group; 20 × 4 = 80). The different doses (50, 100 and 200 μM) of all DKPs were treated with transplanted embryos. Before DKPs treatment, the initial fluorescence intensity of transplanted cancer cells was measured at 0 hour post-injection (hpi). At the end of experiment 24, 48, and 72 hpi, all embryos were selected from each well and were photographed under an inverted fluorescence microscope (Nikon Eclipse TE2000-U, Tokyo, Japan). The following acquisition parameters were employed over time: Image scaling: Low = 130; High = 2,900; Exposure Time = 100 ms and Binning = 1. The fluorescence intensity of the tumor in the embryos was measured and the percentage of tumor growth inhibition was calculated according to the following formula:

$${\text{Inhibition}}\;{\text{rate}}(\% ) = \frac{{{\text{Z}}\;({\text{Negative}}\;{\text{control}}) - {\text{Z}}\,({\text{Treatment}})}}{{{\text{Z}}\,({\text{Negative}}\;{\text{control}})}} \times 100\%$$

### Statistical analysis

Results were expressed as mean ± standard deviation (SD). Data were analyzed one-way analysis of variance (ANOVA), followed by Student’s two-tailed *t*-test for comparison between two groups and Dunnett’s t-test was used when the data involved three or more groups. A value of *p* < 0.05 was considered statistically significant. All statistical analyses and inhibitory concentration (IC_50_) were determined by graph-pad prism statistical software (San Diego, CA, USA).

## Supplementary information

Supplementary Information.
