# Supplementary material for: Induction of mitochondria-mediated apoptosis and suppression of tumor growth in zebrafish xenograft model by cyclic dipeptides identified from Exiguobacterium acetylicum
Source: Sci Rep. 2020 Aug 13;10:13721. doi: 10.1038/s41598-020-70516-x (PMC7426938; doi:10.1038/s41598-020-70516-x)
Supplement: Supplementary file 1 — Supplementary Information. [file 41598_2020_70516_MOESM1_ESM.pdf]

Supplementary Information

Induction of mitochondria-mediated apoptosis and suppression of tumor growth in zebrafish xenograft model by cyclic dipeptides identified from *Exiguobacterium acetylicum*

Sekar Jinendiran[1], Weilin Teng[2], Hans-Uwe Dahms[3,4**], Wangta Liu[2], Vinoth Kumar Ponnusamy[5], Charles Chien-Chih, Chiu[2], B.S. Dileep Kumar[6*], and Natesan Sivakumar[1*]

[1] Department of Molecular Microbiology, School of Biotechnology, Madurai Kamaraj University, Madurai-625021, India.

[2] Department of Biotechnology, Kaohsiung Medical University, Kaohsiung-80708, Taiwan.

[3] Department of Biomedical Science and Environmental Biology, Kaohsiung Medical University, Kaohsiung-80708, Taiwan.

[4] Department of Marine Biotechnology and Bioresources, National Sun Yat-sen University, Kaohsiung-80424, Taiwan.

[5] Department of Medicinal Applied Chemistry, Kaohsiung Medical University, Kaohsiung-80708, Taiwan.

[6] Agro-Processing and Technology Division, CSIR-National Institute for Interdisciplinary Science and Technology, Thiruvananthapuram-695019, India.

*** Corresponding author:**

E-mail address: sivamku.ac@gmail.com (N. Sivakumar)

E-mail address: kumardileep@niist.res.in (B.S. Dileep Kumar)

****Co-corresponding author:**

E-mail address: hansd@kmu.edu.tw

<u>NMR spectra of isolated compounds from *E. acetylicum* S01</u>

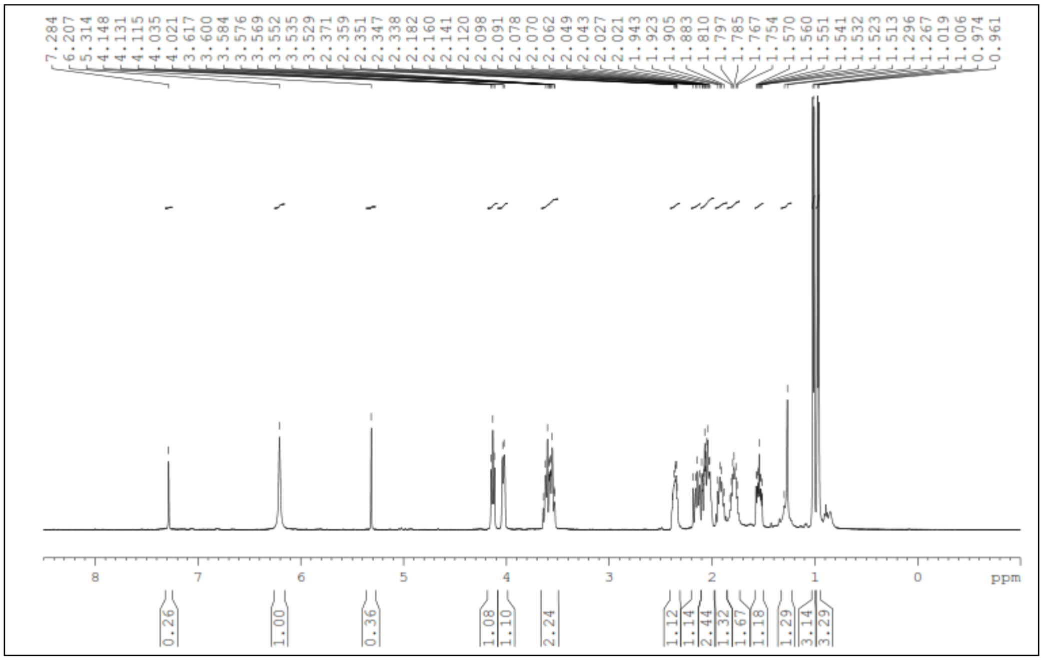

Figure S1A. ^{1}H NMR spectrum of DKP-1 (500 MHz; $CDCl_3$)

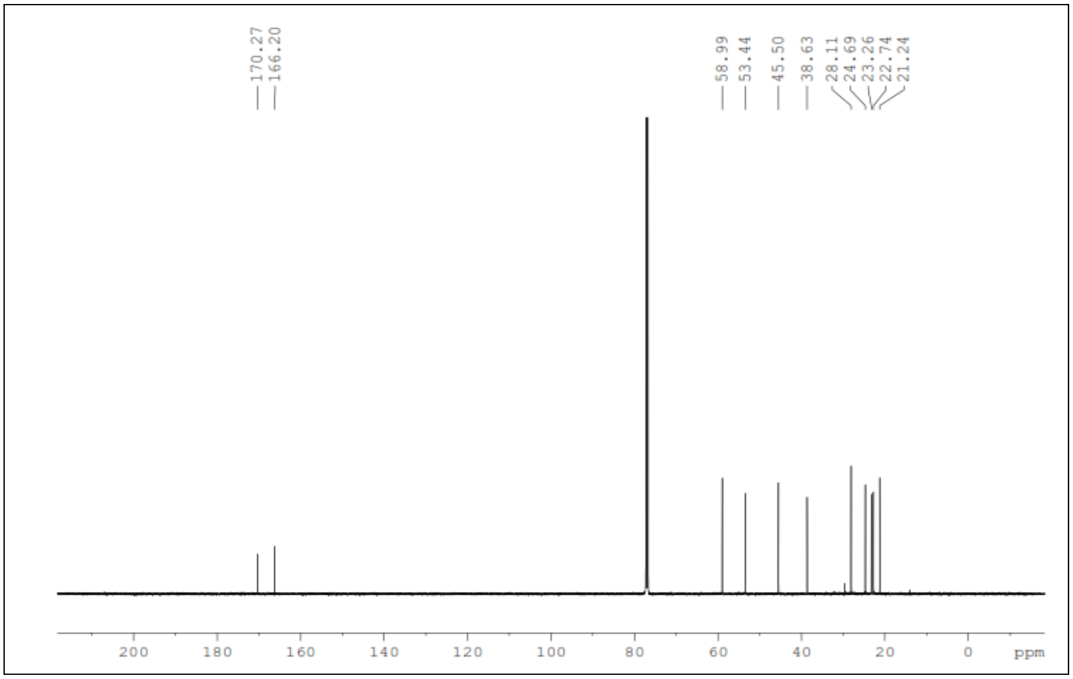

Figure S1B. ^{1}C NMR spectrum of DKP-1 (500 MHz; $CDCl_3$)

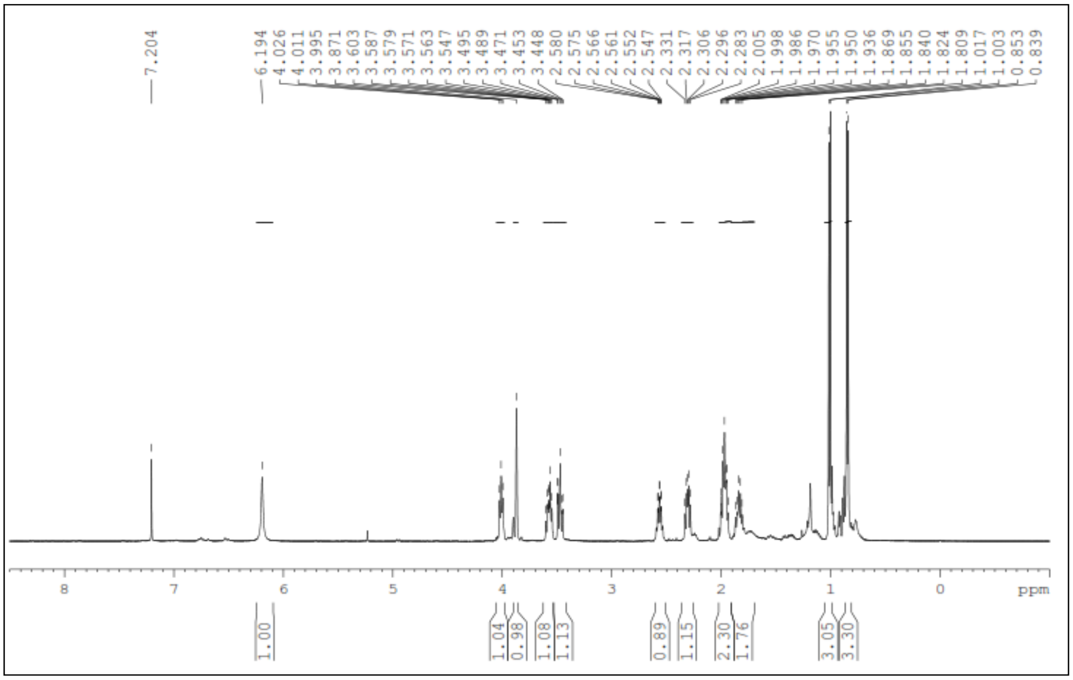

Figure S1C. ^{1}H NMR spectrum of DKP-2 (500 MHz; CDCl$_3$)

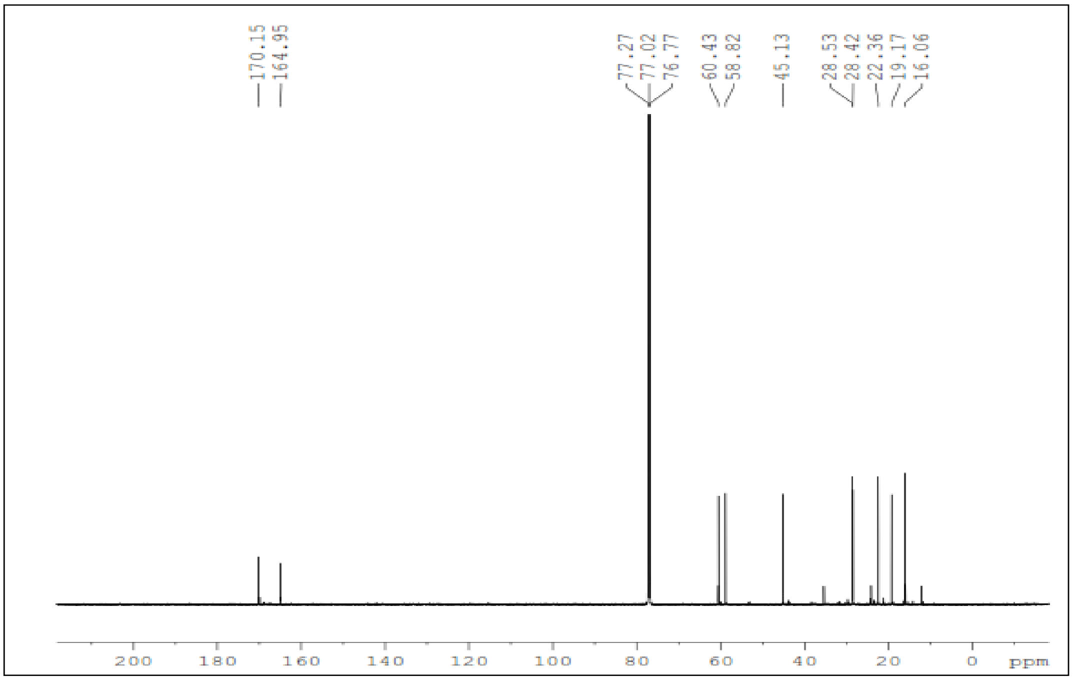

Figure S1D. ^{1}C NMR spectrum of DKP-2 (500 MHz; CDCl$_3$)

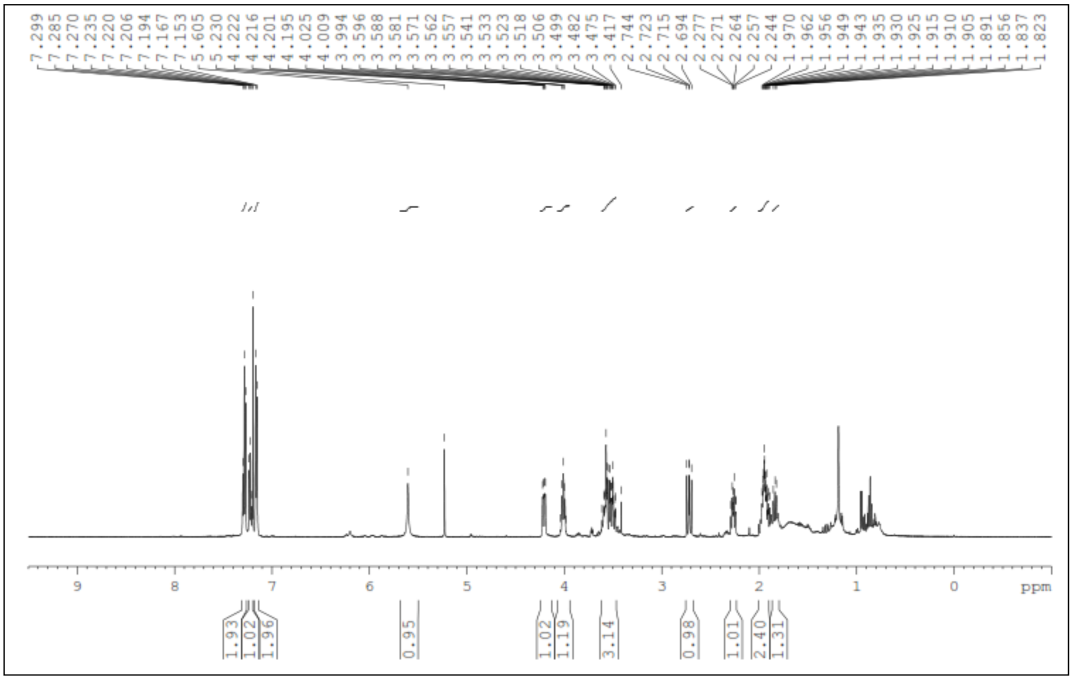

Figure S1E. ^{1}H NMR spectrum of DKP-3 (500 MHz; $CDCl_3$)

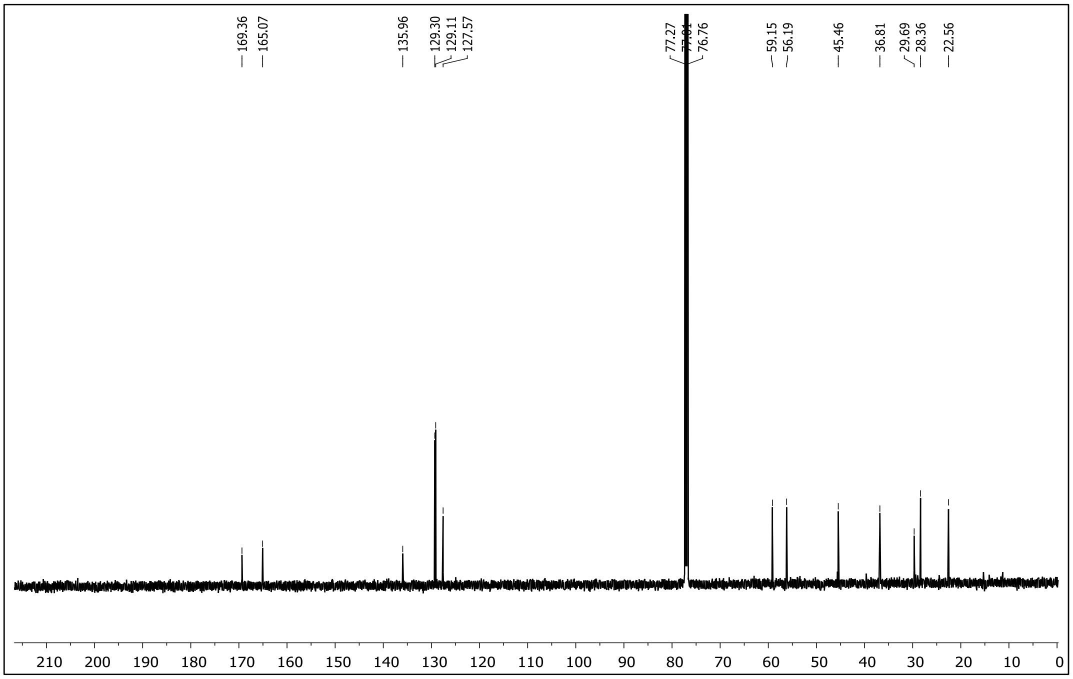

Figure S1F. ^{1}C NMR spectrum of DKP-3 (500 MHz; $CDCl_3$)

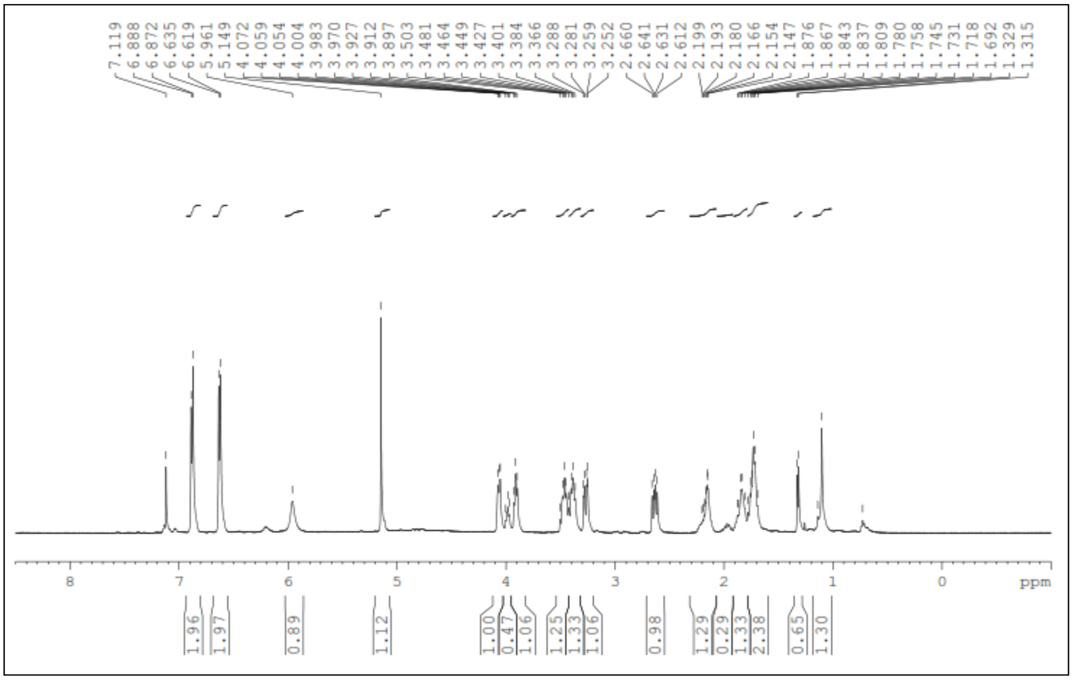

Figure S1G. ^{1}H NMR spectrum of DKP-4 (500 MHz; $CDCl_3$)

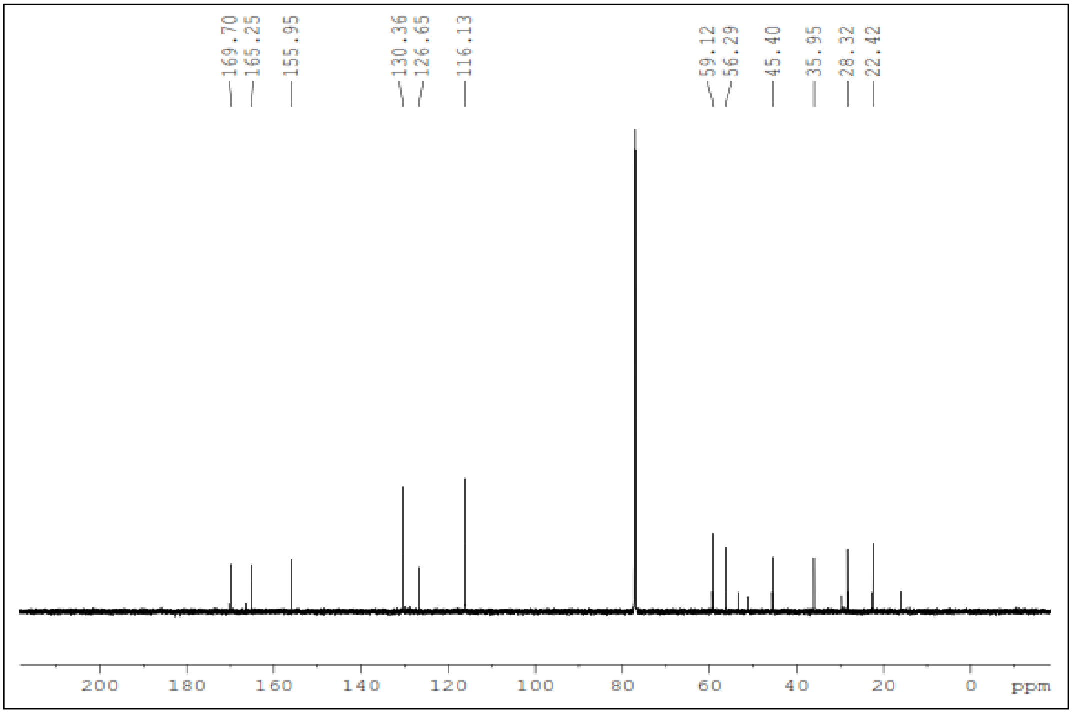

Figure S1H. ^{1}C NMR spectrum of DKP-4 (500 MHz; $CDCl_3$)

ESI-MS spectra of isolated cyclic dipeptides from *E. acetylicum* S01

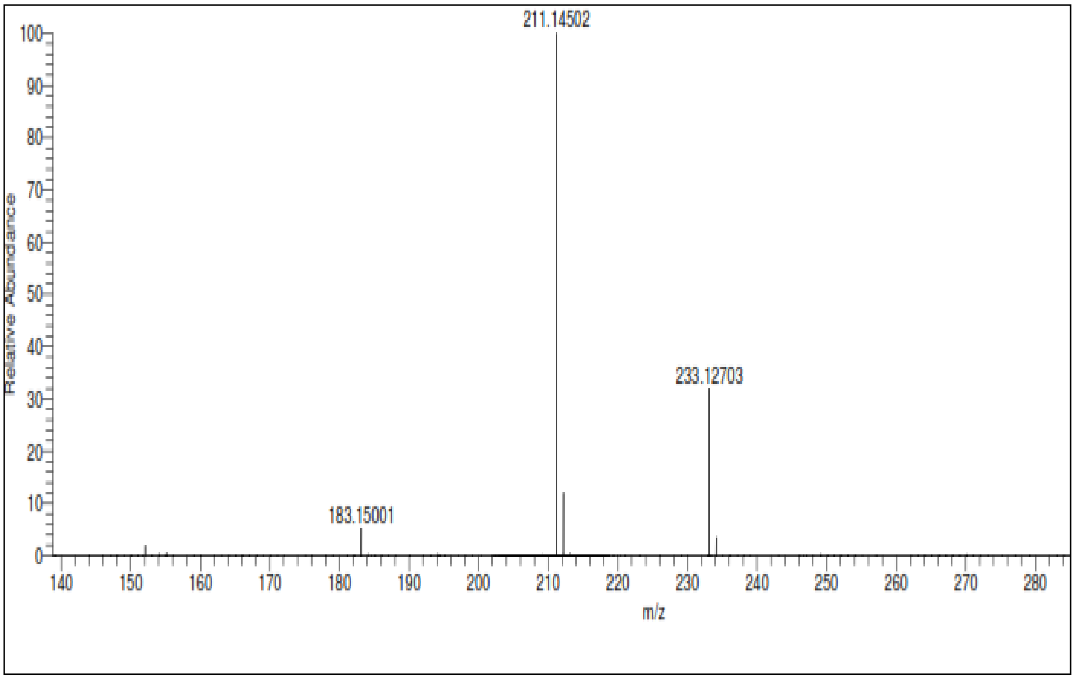

Figure S2A. Mass spectrum (ESI-MS) of DKP-1 (M+1)

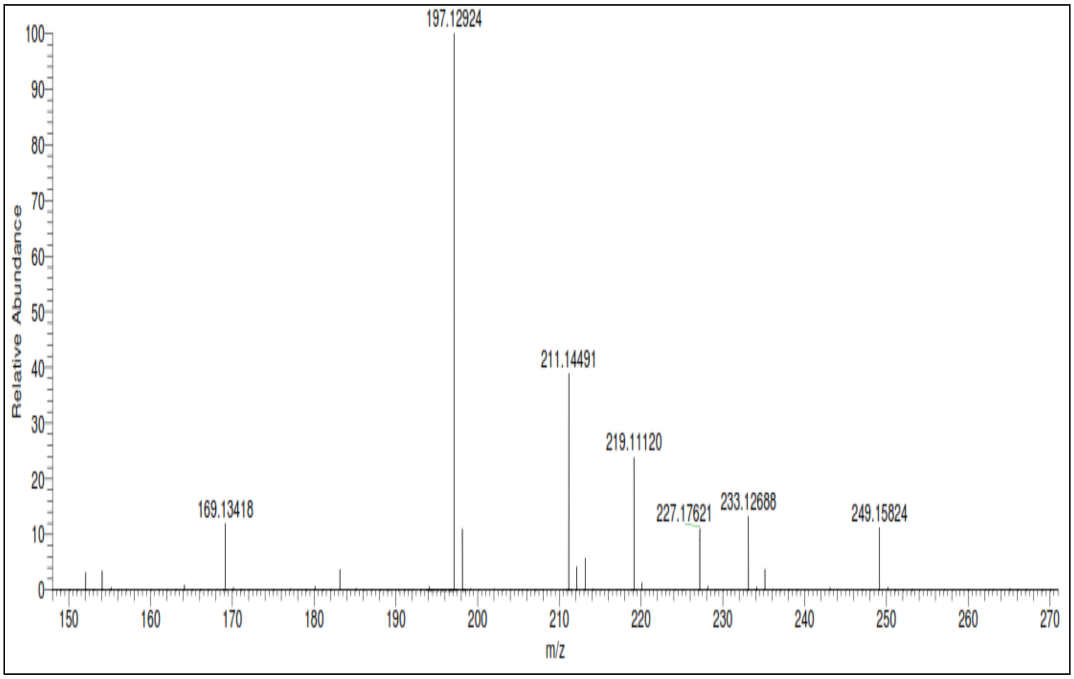

Figure S2B. Mass spectrum (ESI-MS) of DKP-2 (M+1)

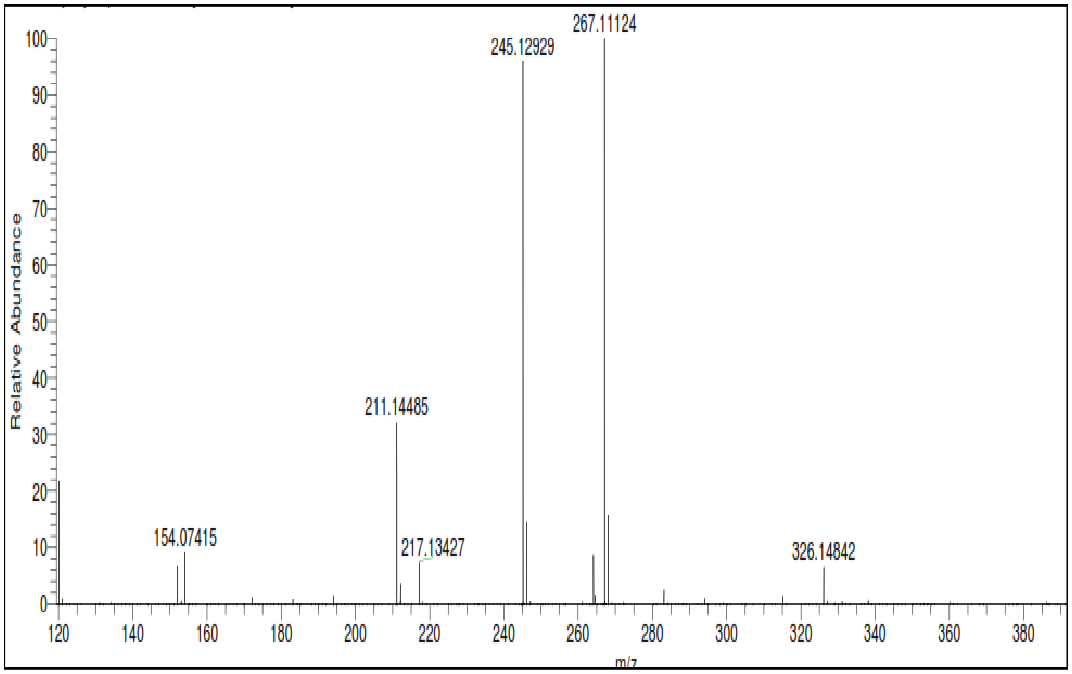

Figure S2C. Mass spectrum (APCI) of DKP-3 (M+1)

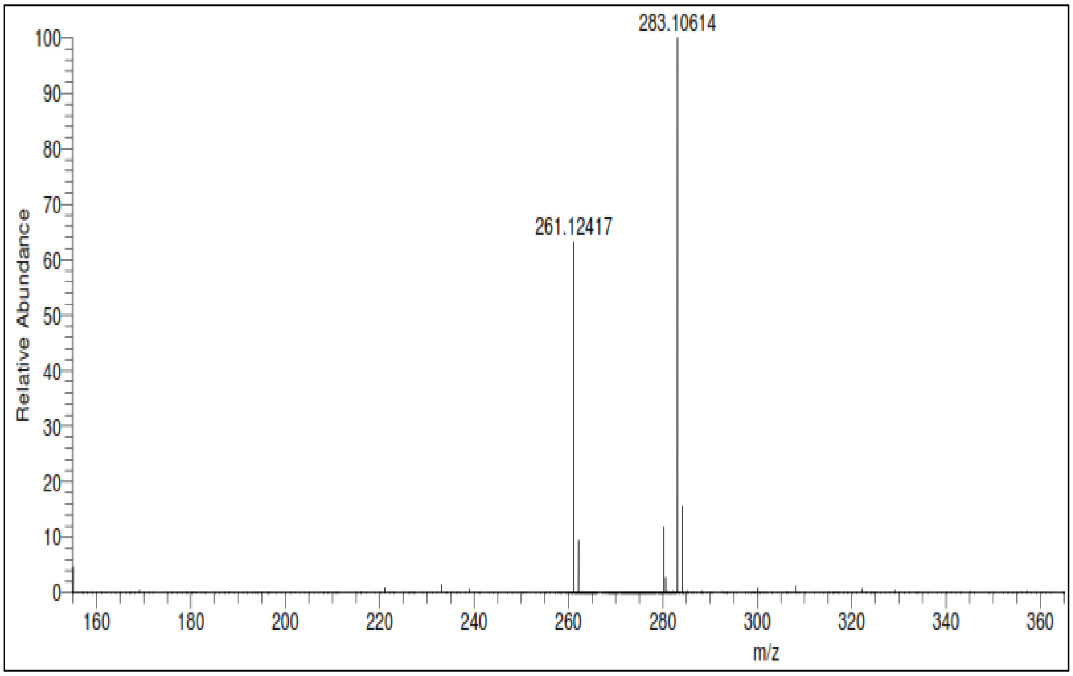

Figure S2D. Mass spectrum (ESI-MS) of DKP-4 (M+1)

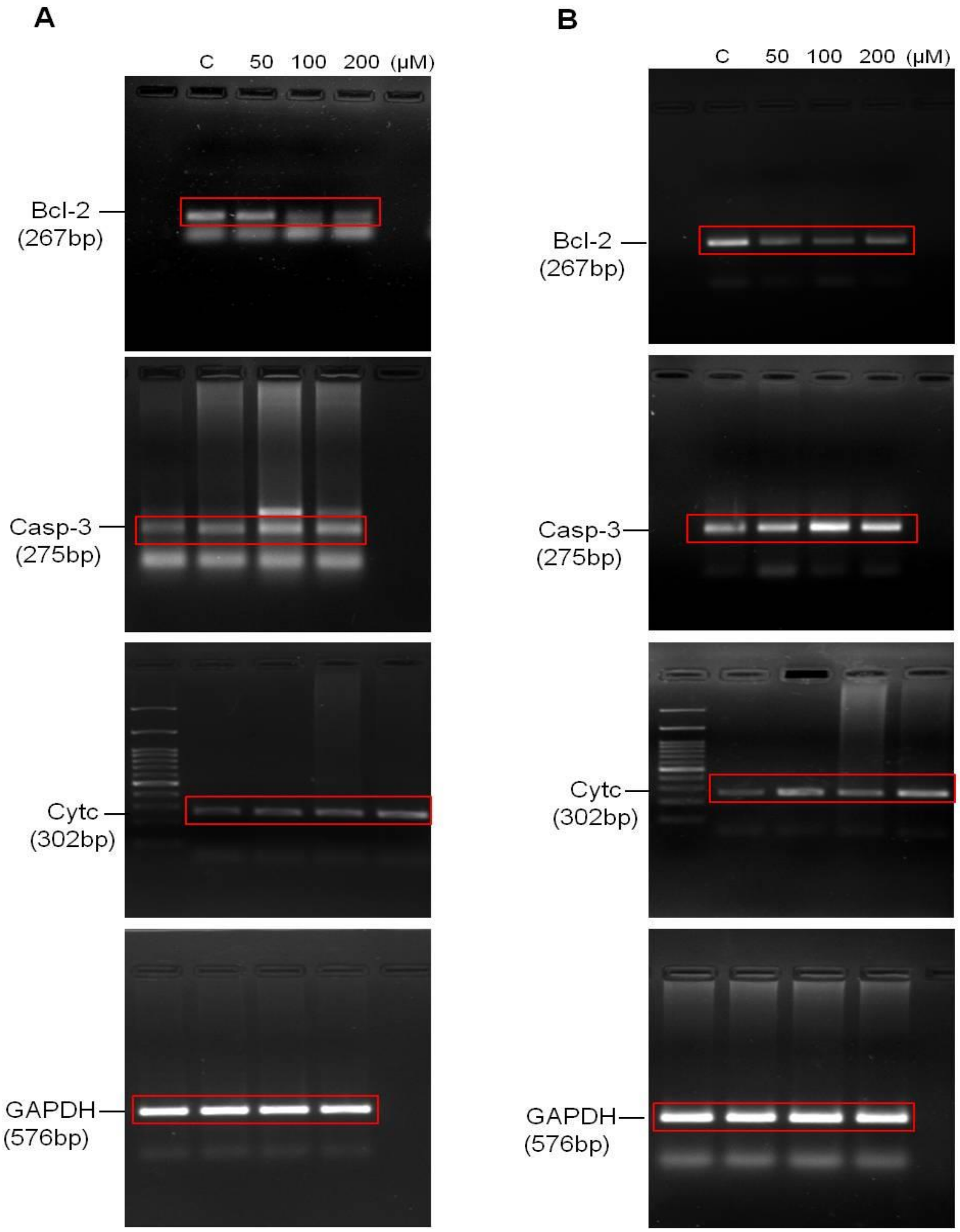

Figure S3A-B. The figure showed the full original and un-cropped images for the agarose gels of Fig. 4A and Fig. 4B displayed in the text and results. The identification of the Bcl-2, Casp-3, Cytc, and GAPDH bands was based on the expected amplicon size (basepair; bp). The GAPDH was used as the cDNA loading control, respectively.

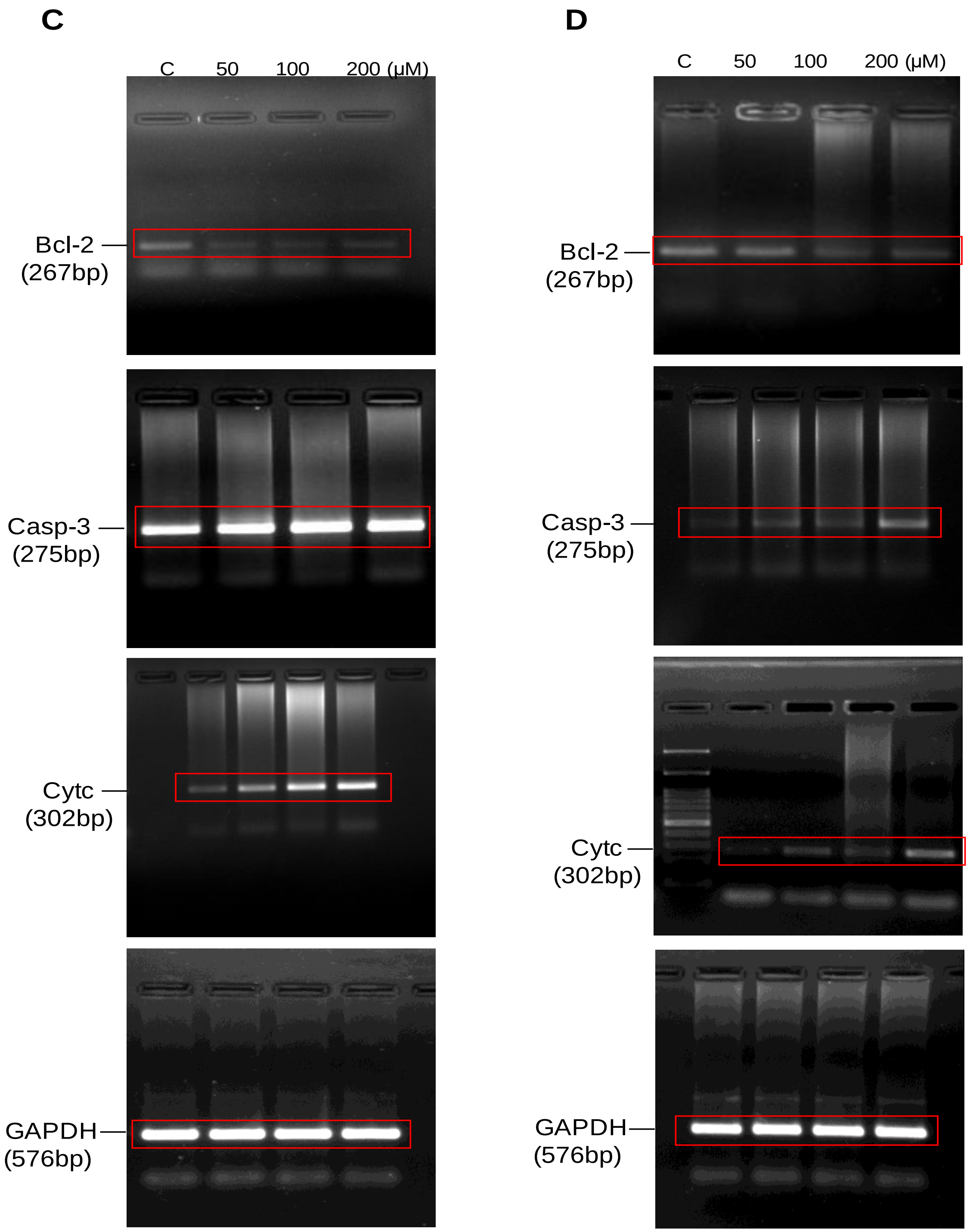

Figure S3C-D. The figure showed the full original and un-cropped images for the agarose gels of Fig. 4C and Fig. 4D displayed in the text and results. The identification of the Bcl-2, Casp-3, Cytc, and GAPDH bands was based on the expected amplicon size (basepair; bp). The GAPDH was used as the cDNA loading control, respectively.

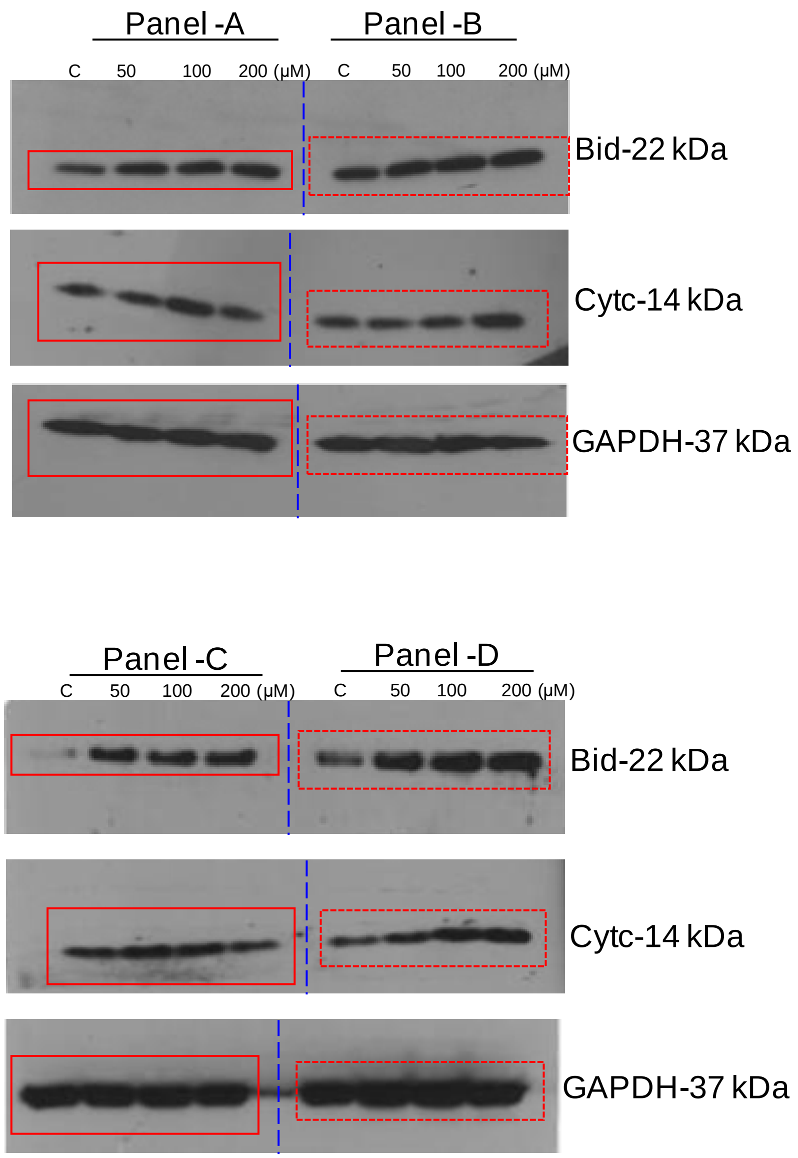

Figure S4 Panel-A-D. The figure showed the full original and un-cropped images for the western blots of Fig. 5A-D displayed in the text and results. The identification of the Bid, Cytc and GAPDH bands were based on the expected molecular weight. The GAPDH was used as the protein loading control, respectively.

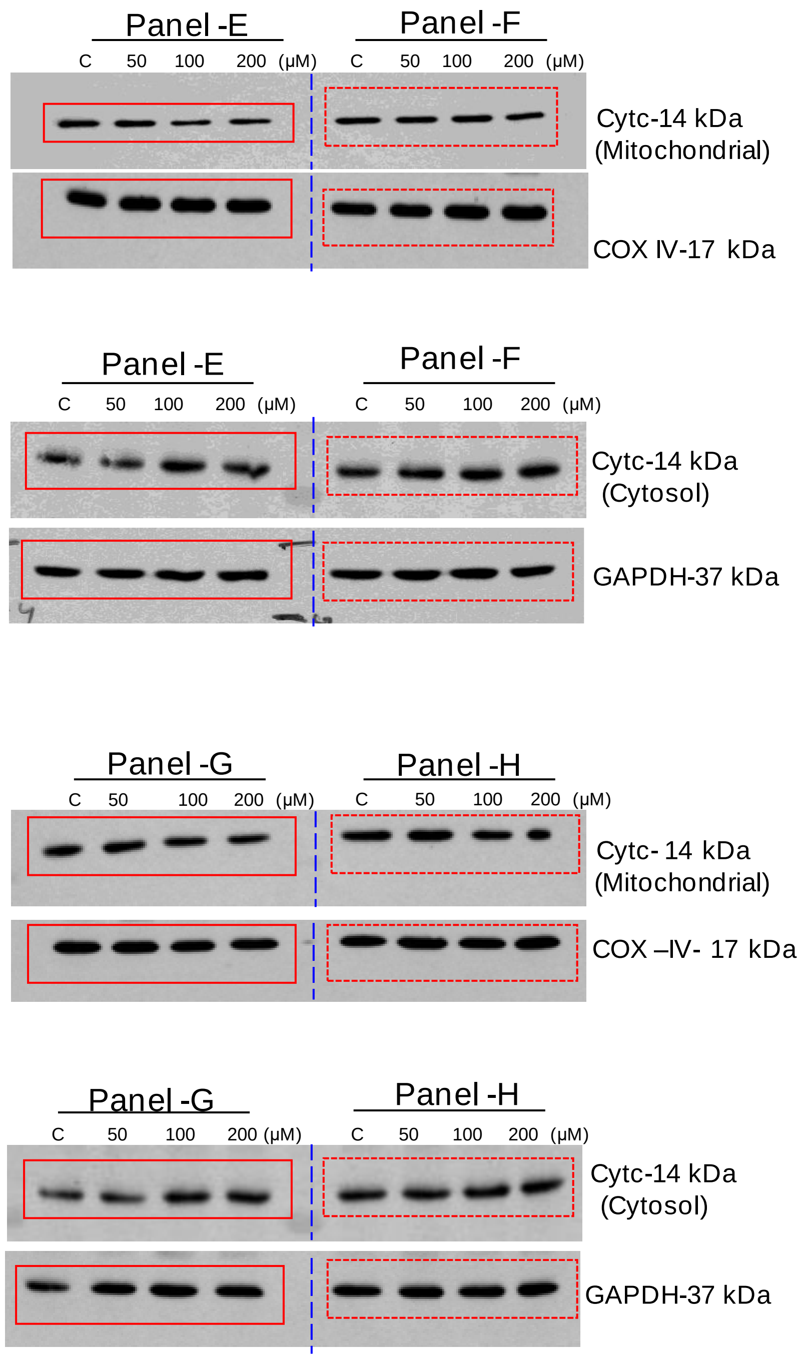

Figure S4 Panel-E-H. The figure showed the full original and un-cropped images for the blots of Fig. 5E-H displayed in the text and results. The identification of the Cytc, COX IV and GAPDH bands were based on the expected molecular weight. The COX IV and GAPDH were used as the cytosolic and mitochondrial protein loading control, respectively.

A: Antitumor activity of DKP-1

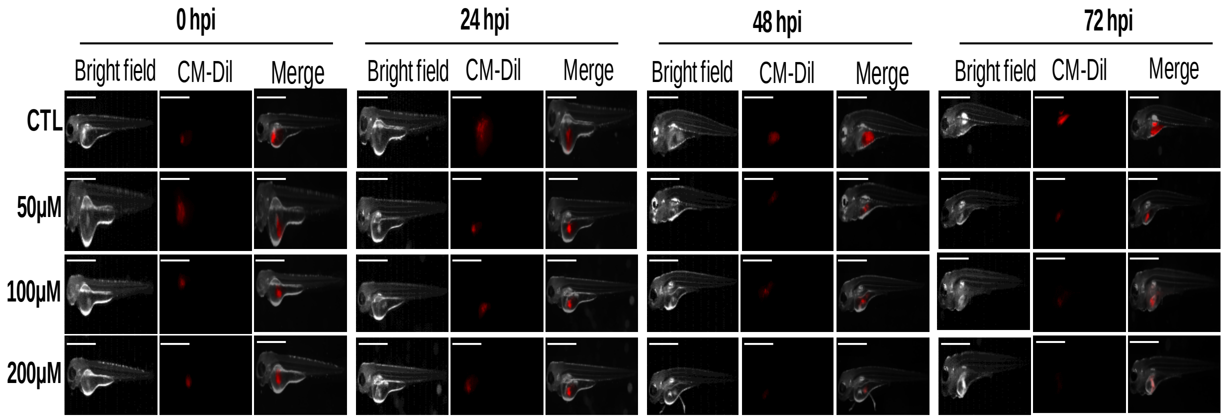

B: Antitumor activity of DKP-2

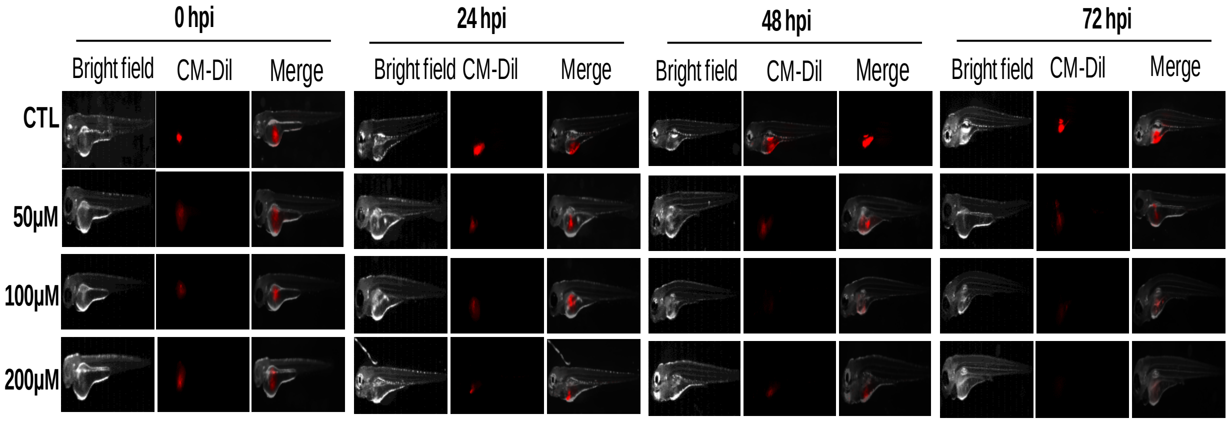

C: Antitumor activity of DKP-3

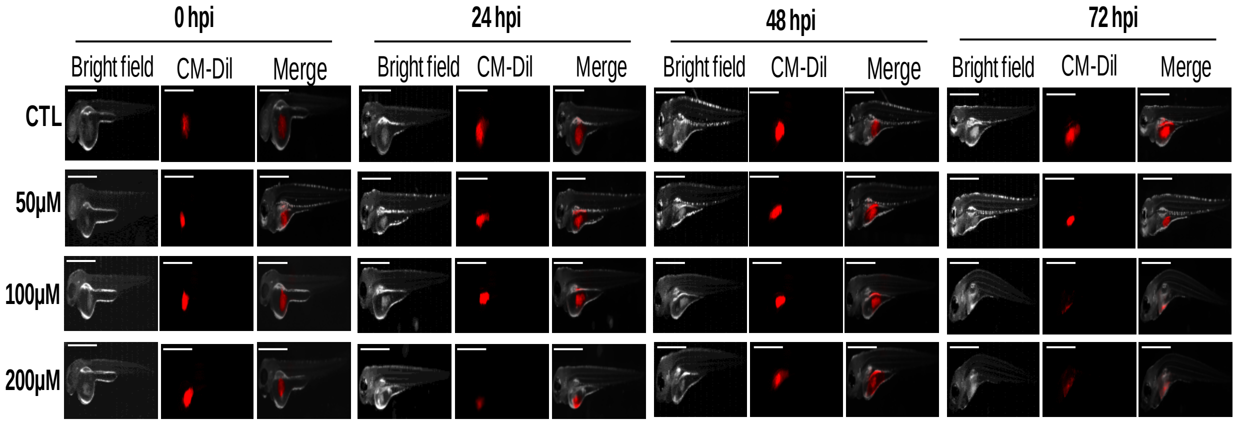

D: Antitumor activity of DKP4

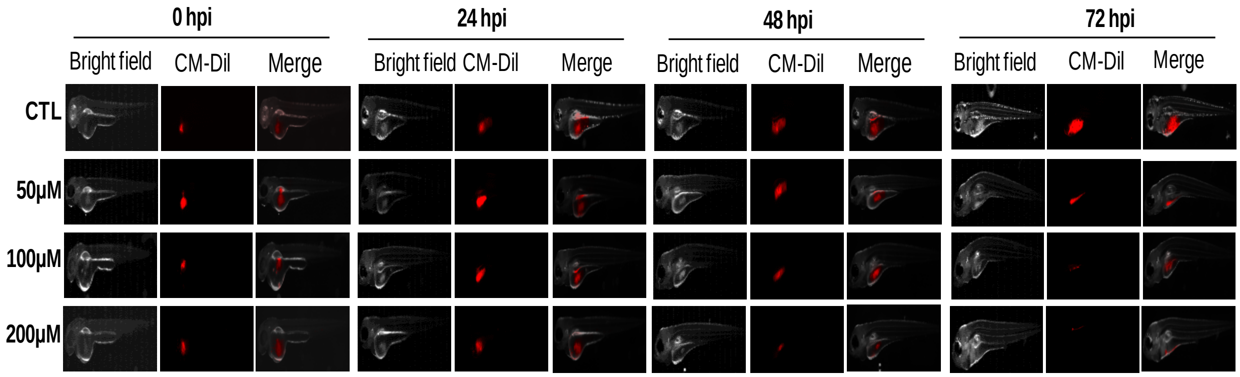

Figure S5. Antitumor effects of four cyclic dipeptides in a zebrafish xenograft model. The figure showed the full original and un-cropped images for the zebrafish/tumor xenograft images of Fig. 7A-D displayed in the text and results. On 2 dpf, CM-Dil labelled HT-29 xenografted embryos in DKPs treated and un-treated larvae for 72 h. Representative images for bright field and fluorescent images showing DKPs treated and un-treated HT-29 xenograft zebrafish larvae at 0 hpi, 24 hpi, 48 hpi and 72 hpi, respectively. CTL – Control; Scale bar = 200 µm.

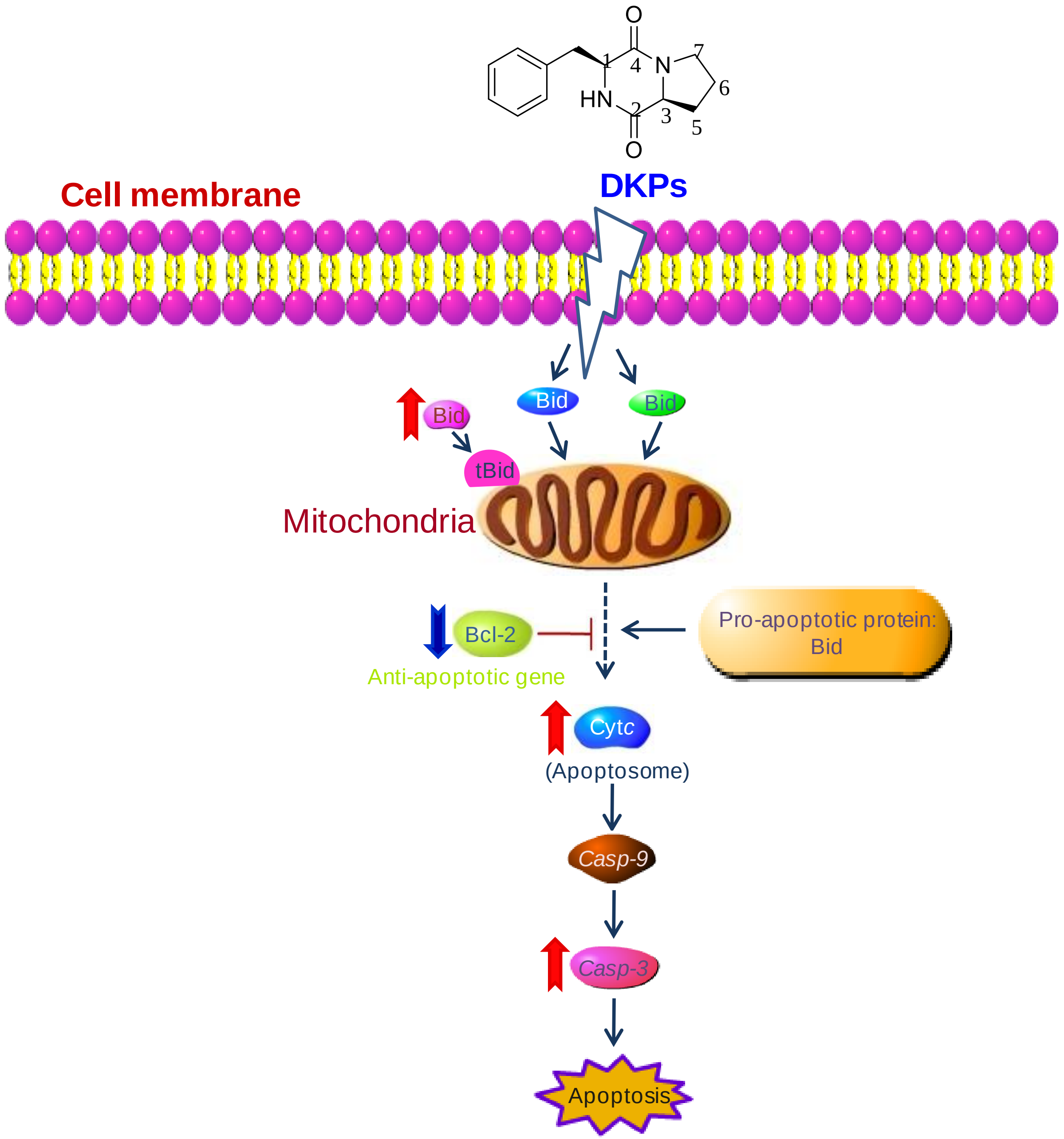

Figure S6. Proposed schematic diagram of the effects of DKPs induces apoptosis in HT-29 cells. When cells are damaged by DKPs treatment, Cytc was released from mitochondria to cytosol. The released Cytc triggers a series of processes that in turn activate caspase-9, which then activates caspase-3. Activated caspase-3 executes apoptosis of HT-29 cells. Moreover, the up-regulation of apoptotic (Cytc, caspase-3 and Bid) and down-regulation of anti-apoptotic (Bcl-2) markers would increase apoptosis in HT-29 cells by mitochondria-mediated apoptosis pathway.
